# Predictors of Chemosensitivity in Triple Negative Breast Cancer: An Integrated Genomic Analysis

**DOI:** 10.1371/journal.pmed.1002193

**Published:** 2016-12-13

**Authors:** Tingting Jiang, Weiwei Shi, Vikram B. Wali, Lőrinc S. Pongor, Charles Li, Rosanna Lau, Balázs Győrffy, Richard P. Lifton, William F. Symmans, Lajos Pusztai, Christos Hatzis

**Affiliations:** 1 Department of Medicine, Yale School of Medicine, Yale University, New Haven, Connecticut, United States of America; 2 MTA TTK Lendulet Cancer Biomarker Research Group, Research Center for Natural Sciences, Budapest, Hungary; 3 2nd Department of Pediatrics, Semmelweis University, Budapest, Hungary; 4 Department of Genetics, Yale School of Medicine, Yale University, New Haven, Connecticut, United States of America; 5 Department of Pathology, University of Texas MD Anderson Cancer Center, Houston, Texas, United States of America; 6 Yale Cancer Center, New Haven, Connecticut, United States of America; MSKCC, UNITED STATES

## Abstract

**Background:**

Triple negative breast cancer (TNBC) is a highly heterogeneous and aggressive disease, and although no effective targeted therapies are available to date, about one-third of patients with TNBC achieve pathologic complete response (pCR) from standard-of-care anthracycline/taxane (ACT) chemotherapy. The heterogeneity of these tumors, however, has hindered the discovery of effective biomarkers to identify such patients.

**Methods and Findings:**

We performed whole exome sequencing on 29 TNBC cases from the MD Anderson Cancer Center (MDACC) selected because they had either pCR (*n* = 18) or extensive residual disease (*n* = 11) after neoadjuvant chemotherapy, with cases from The Cancer Genome Atlas (TCGA; *n* = 144) and METABRIC (*n* = 278) cohorts serving as validation cohorts. Our analysis revealed that mutations in the AR- and FOXA1-regulated networks, in which BRCA1 plays a key role, are associated with significantly higher sensitivity to ACT chemotherapy in the MDACC cohort (pCR rate of 94.1% compared to 16.6% in tumors without mutations in AR/FOXA1 pathway, adjusted *p* = 0.02) and significantly better survival outcome in the TCGA TNBC cohort (log-rank test, *p* = 0.05). Combined analysis of DNA sequencing, DNA methylation, and RNA sequencing identified tumors of a distinct BRCA-deficient (BRCA-D) TNBC subtype characterized by low levels of wild-type BRCA1/2 expression. Patients with functionally BRCA-D tumors had significantly better survival with standard-of-care chemotherapy than patients whose tumors were not BRCA-D (log-rank test, *p* = 0.021), and they had significantly higher mutation burden (*p <* 0.001) and presented clonal neoantigens that were associated with increased immune cell activity. A transcriptional signature of BRCA-D TNBC tumors was independently validated to be significantly associated with improved survival in the METABRIC dataset (log-rank test, *p* = 0.009). As a retrospective study, limitations include the small size and potential selection bias in the discovery cohort.

**Conclusions:**

The comprehensive molecular analysis presented in this study directly links BRCA deficiency with increased clonal mutation burden and significantly enhanced chemosensitivity in TNBC and suggests that functional RNA-based BRCA deficiency needs to be further examined in TNBC.

## Abstract 2

Through molecular analyses of tissue from triple negative breast cancer patients, Christos Hatzis and colleagues report on a new tumor subtype with possible implications for treatment.

## Author Summary

**Why Was This Study Done?:**

- Identifying chemosensitive triple negative breast cancers (TNBCs) could significantly impact the survival of patients with these difficult to treat cancers until novel targeted therapies become available.
- We hypothesized that genomic somatic aberrations may provide important molecular clues about chemosensitivity in TNBC.
- Our study used a carefully selected cohort of 29 uniformly treated TNBC patients who either achieved pathologic complete response (pCR) or had extensive residual disease after neoadjuvant anthracycline/taxane chemotherapy.

**What Did the Researchers Do and Find?:**

- We sequenced the coding genomic DNA of TNBC tumors and compared the somatic mutations found in the two groups at the two extremes of the chemosensitivity spectrum.
- Our analysis revealed that, although mutations in single genes were not individually predictive, TNBC tumors bearing mutations in genes involved in the androgen receptor (AR) and FOXA1 pathways were much more sensitive to chemotherapy.
- We also found that mutations that lowered the levels of functional *BRCA1* or *BRCA2* RNA were associated with significantly better survival outcomes; we derived a BRCA deficiency signature to define this new, highly chemosensitive subtype of TNBC.
- BRCA-deficient TNBC tumors have a higher rate of clonal mutation burden, defined as more clonal tumors with a higher number of mutations per clone, and are also associated with a higher level of immune activation, which may explain their greater chemosensitivity.

**What Do These Findings Mean?:**

- Mutations in the AR/FOXA1 pathway provide a novel marker for identifying chemosensitive TNBC patients who may benefit from current standard-of-care chemotherapy regimens.
- The newly defined RNA-based BRCA-deficient subtype includes up to 50% of the TNBC tumors that appear to be immune primed, and it would be of interest to investigate combinations of chemotherapy with immunotherapies, which could provide clinical benefit for these patients.
- Although our study showed concordant results in three different datasets, our key findings need to be further validated in a larger, prospectively designed study with archival samples.

## Introduction

Triple negative breast cancer (TNBC) disproportionately affects younger women and women of African ancestry, contributing to health disparities. In the era of personalized cancer therapy, patients with TNBC remain at considerably higher risk of relapse and death than patients with other breast cancer subtypes, due to the aggressive nature of TNBC and the lack of newer targeted therapies [1,2]. TNBC patients typically receive chemotherapy with anthracycline and cyclophosphamide followed by taxane (anthracycline/taxane [ACT]) as standard-of-care treatment. Approximately one-third of patients achieve pathologic complete response (pCR) and have excellent survival, but the remaining patients relapse and eventually die of the disease [3–5]. Identifying those TNBC patients who might benefit from ACT chemotherapy and directing the remaining patients to novel targeted therapies may be an effective strategy with near-term clinical impact for managing TNBC.

Transcriptional signatures developed in the past decade to predict sensitivity to ACT chemotherapy in TNBC have had only partial success [6–8], in part owing to the extensive molecular heterogeneity of the disease [9,10]. A few studies have evaluated whether predictability can be improved by considering a tumor’s somatic genetic aberrations alone [11,12] or in combination with gene expression [13]. Recent next-generation sequencing efforts have identified genes recurrently mutated in TNBC, including *TP53* and *PIK3CA*, but unfortunately have not yielded any new predictive or prognostic clues [12,14]. Among existing markers of chemosensitivity, *BRCA* germline mutation carriers are known to receive greater benefit from platinum-based chemotherapy [15] and poly(ADP-ribose) polymerase (PARP) inhibitors in TNBC and ovarian cancers [16–18], but it is unclear whether patients with these tumors also benefit from ACT chemotherapy. Furthermore, higher prevalence of tumor-infiltrating lymphocytes (TILs) has been associated with better prognosis [19–22], irrespective of the chemotherapy administered, and also with a higher rate of pCR to neoadjuvant anthracycline-based chemotherapy in TNBC [23]. Gene expression signatures that capture immune or stromal characteristics have shown promising prognostic performance [24,25].

Chemosensitive or chemoresistant phenotypes can arise in genomically heterogeneous cancers through diverse molecular mechanisms, resulting in weaker biomarker–phenotype associations at the population level and confounding the discovery of prognostic and predictive biomarkers associated with response. Moreover, mutations alone may not be generally predictive, as gene expression levels are also modulated through non-genetic mechanisms, such as epigenetic silencing, aberrant transcription, and allele-specific expression [26–28]. Broader tumor genomic metrics that capture the extent and diversity of genetic heterogeneity within single tumors, such as overall mutation load and clonality, could be promising biomarkers and have been reported to be associated with patients’ clinical outcome in melanoma and in head and neck cancers [29,30]. There is also renewed interest in the enhanced innate immune response triggered by neoantigens from mutated cancer cell DNA, especially in tumors with mismatch repair deficiency [31], a mechanism that may provide a potential link between BRCA-deficiency-related chemosensitivity and the protective effect of TILs in TNBC.

In this study, we present a comprehensive assessment of chemosensitivity and resistance to ACT in TNBC using whole exome sequencing (WES) to identify tumor genomic aberrations that are potentially predictive of response. We used a TNBC cohort from the MD Anderson Cancer Center (MDACC) consisting of both extremely sensitive and highly recalcitrant tumors to identify mutations in specific genes or pathways that may indicate response or resistance to ACT chemotherapy in TNBC, and validated our findings in a larger TNBC cohort from The Cancer Genome Atlas (TCGA) project. We used integrated whole exome, DNA methylation, copy number variation, and RNA sequencing (RNAseq) data to expand the definition of TNBC subgroups associated with better outcome.

## Methods

### Sample Collection and Datasets

#### MDACC cohort

This study was designed as a follow-up from a previous study [6] and did not have a specific prospective analysis plan. Twenty-nine TNBC samples were selected from a prospectively collected tissue bank of fine needle aspiration biopsies obtained prior to preoperative ACT chemotherapy at the MDACC [6] to represent two extreme response groups: patients achieving pCR (*n* = 18) and patients with extensive residual disease (RD) (*n* = 11) at surgery. All samples from patients in the two response groups from the original cohort of 205 TNBC cases that had tissue available for DNA extraction were included. Patients provided written informed consent in the original study [6]. Clinicopathologic information for these cases is provided in Table 1. This discovery cohort was used to identify mutations associated with response or resistance to ACT chemotherapy.

**Table 1:** Clinical characteristics of MDACC cohort.

Sample ID	RCB Class	Tumor Grade	Tumor Size (T-Stage)	Nodal Status (N-Stage)	AJCC Stage	ER Status	PR Status	HER2 Status
324_012_012	pCR	3	T2	N1	IIB	Neg	Neg	Neg
367_005_005	pCR	3	T4	N1	IIIB	Neg	Neg	Neg
402_006_006	pCR	3	T4	N2	IIIB	Neg	Neg	Neg
485_004_004	pCR	3	T4	N2	IIIB	Neg	Neg	Neg
524_006_006	pCR	3	T2	N1	IIA	Neg	Neg	Neg
557_012_012	pCR	3	T2	N3	IIIB	Neg	Neg	Neg
658_004_004	pCR	3	T3	N3	IIIB	Neg	Neg	Neg
661_005_005	pCR	3	T2	N1	IIB	Neg	Neg	Neg
665_006_006	pCR	3	T2	N1	IIB	Neg	Neg	Neg
692_004_004	pCR	2	T2	N3	IIIB	Neg	Neg	Neg
696_005_005	pCR	3	T2	N1	IIB	Neg	Neg	Neg
735_012_012	pCR	3	T2	N1	IIB	Neg	Neg	Neg
805_005_005	pCR	2	T2	N1	IIB	Neg	Neg	Neg
M211_006_006	pCR	3	T3	N2	IIIA	Neg	Neg	Neg
M571_004_004	pCR	3	T2	N1	IIB	Neg	Neg	Neg
M635_005_005	pCR	3	T1	N3	IIIC	Neg	Neg	Neg
M750_006_006	pCR	3	T2	N2	IIIA	Neg	Pos	Neg
M792_012_012	pCR	3	T3	N2	IIIA	Neg	Neg	Neg
757_004_004	RCB-I	2	T2	N3	IIIB	Neg	Neg	Neg
348_004_004	RCB-II	3	T3	N1	IIIA	Neg	Neg	Neg
494_005_005	RCB-II	3	T2	N0	IIA	Neg	Neg	Neg
681_012_012	RCB-II	3	T3	N0	IIB	Neg	Neg	Neg
219_005_005	RCB-III	3	T4	N3	IIIB	Neg	Neg	Neg
229_006_006	RCB-III	3	T3	N1	IIIA	Neg	Neg	Neg
612_006_006	RCB-III	3	T2	N2	IIIA	Neg	Neg	Neg
732_006_006	RCB-III	3	T2	N2	IIIA	Neg	Neg	Neg
M345_012_012	RCB-III	3	T4	N2	IIIB	Neg	Neg	Neg
125_004_004	RD	3	T1	N1	IIA	Neg	Neg	Neg
406_012_012	RD	3	T2	N2	IIIA	Neg	Neg	Neg

All patients received 12 weekly doses of paclitaxel followed by four cycles of fluorouracil, doxorubicin, and cyclophosphamide and then surgery.

AJCC, American Joint Committee on Cancer; ER, estrogen receptor; HER2, human epidermal growth factor receptor 2; Neg, negative; pCR, pathologic complete response; Pos, positive; PR, progesterone receptor; RCB, residual cancer burden; RD, residual disease (RCB unknown).

#### TCGA cohort

**Fig 1:**
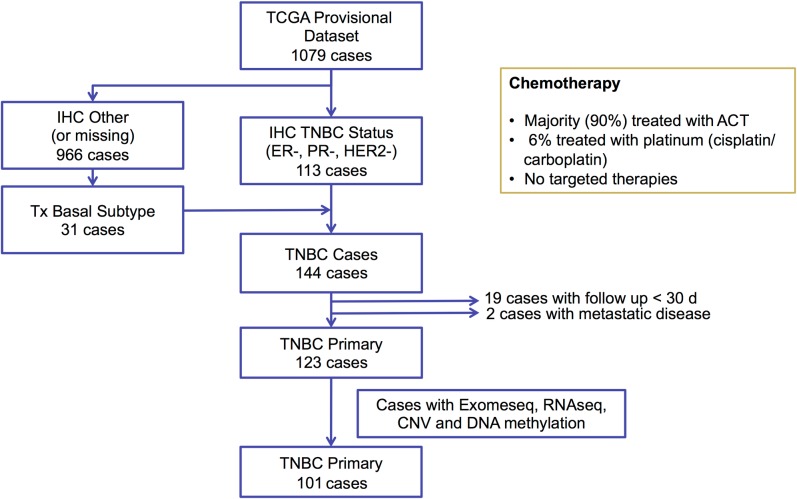
Diagram for selecting triple negative breast cancer samples from the TCGA breast cancer dataset. ACT, anthracycline/taxane; CNV, copy number variation; ER, estrogen receptor; Exomeseq; exome sequencing; IHC, immunohistochemistry; PR, progesterone receptor; RNAseq, RNA sequencing; TCGA, The Cancer Genome Atlas; TNBC, triple negative breast cancer.

We selected 144 TNBC samples from the TCGA breast cancer cohort (https://gdc-portal.nci.nih.gov/projects/TCGA-BRCA) based on estrogen receptor (ER), progesterone receptor (PR), and HER2 immunohistochemistry (IHC) status, when available, or basal-like PAM50 breast cancer subtype (https://www.synapse.org/#!Synapse:syn1700525) if IHC was missing (Fig 1). We excluded 19 cases because of inadequate follow up (<30 d) and two cases with metastatic disease, resulting in 123 TNBC cases. WES-derived somatic and germline variants were available for 102 of these cases. In addition to WES, RNAseq V2 normalized gene expression and DNA methylation (Infinium HumanMethylation450 BeadChip; Illumina) data were available for 101 of these cases and were accessed from the TCGA Data Portal (https://gdc-portal.nci.nih.gov/projects/TCGA-BRCA). Copy number variation data from SNP arrays were accessed from the Broad GDAC Firehose (http://gdac.broadinstitute.org/). Variant allele frequency of somatic mutations was obtained from exome sequencing alignment and RNAseq alignment data from the Cancer Genomics Hub (https://cghub.ucsc.edu/). In addition, we accessed the curated mutations of the overlapped TCGA samples to perform mutation signature analysis [32]. Predicted neoantigens for the same 102 TNBC cases from the TCGA cohort were directly obtained from a previous study [33]. The majority of the TCGA TNBC cases (90%) received ACT chemotherapy, 6% received platinum-based therapies, and none received targeted therapies (Fig 1). The TCGA cohort was used in analyses to validate mutations identified in the MDACC discovery cohort and their association with survival outcomes. This dataset was also used to define the BRCA-deficient (BRCA-D) subtype based on wild-type (WT) BRCA transcript expression and to derive a transcriptional signature for this subtype. We also used this dataset to analyze the association of BRCA deficiency with clonal mutation burden (CMB) and immune activation.

#### METABRIC cohort

We identified 278 basal-like breast cancer cases (because receptor status determined by IHC was not available for all cases) treated with chemotherapy from the METABRIC (Molecular Taxonomy of Breast Cancer International Consortium) dataset. The clinical outcomes and normalized gene expression data generated on the Illumina HumanHT-12 platform were accessed from https://sagebionetworks.jira.com/wiki/display/BCC. Probes from intronic gene regions were excluded from the analysis. We used this cohort as independent validation that patients with BRCA-D TNBC tumors selected based on the transcriptional signature have improved survival outcomes.

### Whole Exome Sequencing and Variant Calling

After DNA extraction from the fine needle aspiration biopsies, 1 μg of genomic DNA was sheared to a mean fragment length of about 140 bp. A NimbleGen human solution-capture exome array (SeqCap EZ v2) was used to capture the exomes of tumor samples, using a procedure modified from the manufacturer’s instructions. The library was sequenced on an Illumina HiSeq 2000 platform in paired-end 75-cycles mode at the Yale Center for Genome Analysis.

Reads were filtered by Illumina CASAVA 1.8.2 software, trimmed at the 3′ end using FASTX v0.0.13, and aligned to the human reference genome (GRCh37) by Burrows-Wheeler Aligner v0.7.5a, and PCR duplicates were removed using the MarkDuplicates (Picard) algorithm. Local realignment around putative and known insertion/deletion (INDEL) sites and base quality recalibration were performed using RealignerTargetCreator (Genome Analysis Toolkit v3.1.1). MuTect v1.1.4 and Strelka v1.0.14 were used to call somatic single nucleotide variants and INDELs, respectively, with an in-house pooled normal reference obtained from ten normal blood DNA samples sequenced using the same protocol. Mutations in the Single Nucleotide Polymorphism Database (dbSNP build 138; https://www.ncbi.nlm.nih.gov/SNP/; variants not flagged as somatic or clinical or as having a minor allele frequency of <1%), the NHLBI Exome Sequencing Project (ESP6500; http://evs.gs.washington.edu/evs_bulk_data/ESP6500SI-V2-SSA137.GRCh38-liftover.snps_indels.vcf.tar.gz), the 1000 Genomes Project (http://www.1000genomes.org/), and Exome Aggregation Consortium dataset (ExAC release 0.1; ftp://ftp.broadinstitute.org/pub/ExAC_release/release0.1) were excluded as putative germline sequence alterations. Furthermore, mutations were also excluded if the ratio of the mutant allele frequency (MAF) in tumor versus the pooled normal was less than five or if the MAF was 0.45–0.55. Recurrent COSMIC (v64) variants (*n* ≥ 5) and ClinVar annotated variants (http://www.ncbi.nlm.nih.gov/clinvar/) were whitelisted. All mutations found to have significant association with clinical outcomes were manually visualized in the Integrative Genomics Viewer (https://www.broadinstitute.org/igv/) to filter potential false positive calls. Raw WES data and mutation calls from the MDACC TNBC cohort are deposited in the Sequence Read Archive (accession ID SRP063902; http://trace.ncbi.nlm.nih.gov/Traces/sra/?study=SRP063902).

### Genomic Biomarker Analyses

#### Gene level

For the MDACC cohort, we applied the MutSigCV algorithm [34] to detect mutations in driver genes occurring above the background mutation rate (MR) with false discovery rate < 10%. Only significant non-silent mutations were aggregated at the gene level to test for association with clinical outcome.

#### Pathway level

We collected 714 canonical pathways from the National Cancer Institute Pathway Interaction Database (http://pid.nci.nih.gov/) and BioCarta (http://www.biocarta.com/). We applied five algorithms, PhyloP, SIFT, PolyPhen2, MutationTaster, and LRT, to predict the functional impact of mutations. We selected high functional impact single nucleotide variants predicted as deleterious by at least three algorithms and all the INDELs. High functional impact variants were aggregated at the pathway level to test for association with chemotherapy response and distant relapse-free survival.

#### Broad genomic metrics

MR was calculated as the total number of mutations detected, normalized by the length of target exome sequenced with at least 10-fold coverage. The mutant allele tumor heterogeneity (MATH) score for a tumor was calculated as the median absolute deviation divided by the median MAF of all somatic mutations detected in the tumor sample. As previously suggested [30], for the calculation of MATH we used somatic mutation calls with MAF of 0.075 or greater. For these analyses, we started with the exome sequence alignment (BAM) files provided by TCGA and called somatic mutations using MuTect.

### Tumor Clonal Analysis

The R package SciClone [35] was used to infer tumor clonality by clustering variants of similar MAF from a tumor sample. We only selected mutations with at least 10-fold coverage and MAF < 0.6 to exclude mutations that may involve copy number loss. Variants were clustered using a Bayesian binomial mixture model in SciClone with each cluster representing a separate clone in the tumor. The average number of mutations per clone in each sample was calculated as the weighted sum of the number of mutations in each clone multiplied by the clonal proportion estimated by SciClone.

### Mutational Signature Analysis

The trinucleotide loadings for four mutational signatures previously identified in breast cancer, Signature.1B (age associated), Signature.2 (APOBEC), Signature.3 (BRCA), and Signature.6 (mismatch), were downloaded from a previous study [32]. We applied non-negative least squares to estimate the proportion of each signature in each sample, and the signature with the greatest estimated coefficient was designated the dominant signature.

### BRCA Deficiency Analysis

#### Epigenetic silencing of BRCA1

We averaged the beta value of four robust probes (cg19531713, cg19088651, cg08993267, and cg04658354) in the promoter region of BRCA1 from the Infinium HumanMethylation450 Beadchip [36]. BRCA1 DNA methylation was categorized as unmethylated (score < 0.2) or methylated (score > 0.2).

#### Wild-type BRCA1/2 expression quantification and definition of BRCA1/2 deficiency

We downloaded RNAseq data analyzed by pipeline V2 using MapSplice and RSEM (https://wiki.nci.nih.gov/display/TCGA/RNASeq). We used normalized count of BRCA1/2 as the measure of overall expression. In cases harboring a BRCA1/2 mutation, we extracted the BRCA1/2 locus (Assembly GRCh37.p13) from the RNAseq alignment file, quantified the MAF in the mutation position, and then calculated the WT BRCA1/2 expression as:

$$Expression({BRCA1/2}_{WT})=Expression({BRCA1/2}_{Total})(1-MAF).$$.

In cases with multiple BRCA mutations, WT BRCA1/2 expression was based on the mutation with the highest MAF. We extended the definition of BRCA-D status to include (1) at least one deleterious BRCA1/2 mutation or (2) for BRCA WT patients, WT BRCA1/2 expression less than the maximum observed in mutation carriers in the cohort.

#### BRCA-deficient gene expression signature

We developed a BRCA deficiency transcriptional signature to assess the predictive potential of BRCA deficiency status in additional datasets. Differentially expressed genes between BRCA-D and BRCA-normal (BRCA-N) cases in the TCGA TNBC cohort were identified using the Bioconductor limma package [37] (unadjusted *p <* 0.002). Genes with median expression less than 25% of the overall median were excluded. A BRCA deficiency metagene was defined as the mean expression of genes overexpressed in BRCA-D cases minus the mean expression of genes overexpressed in BRCA-N cases. Transcriptional BRCA deficiency status for each case was determined by the median dichotomized metagene score. We used the Kaplan-Meier survival estimator and Cox proportional hazards regression to assess the association of BRCA deficiency metagene expression with overall survival in TNBC cases in the TCGA and METABRIC datasets.

### Statistical Analysis

The association between mutational status (at the gene or pathway level) and pCR rate or overall survival was assessed using the Fisher exact test and the Kaplan-Meier survival estimator, respectively. Wilcoxon rank tests were used to compare characteristics (MR, MATH score, number of neoantigens, and mutation signature) between groups (pCR versus RD, BRCA-D versus BRCA-N).

## Results

### Mutations Associated with Chemosensitivity in Triple Negative Breast Cancer

We sequenced the genomic DNA from 29 cases in two response groups (pCR, *n* = 18; RD, i.e., chemoresistant with moderate or extensive residual cancer burden [38], *n* = 11) using WES (mean nucleotide coverage 150×; more than 90% of target bases had >20× coverage in all samples). Most detected somatic mutations were not recurrent, and only the MR of *TP53* was significantly above background across all samples (false discovery rate < 0.1 using MutSigCV [34]). Twenty-two of 29 tumors (76%) carried non-silent *TP53* mutations (S1 Fig), but there was no evidence of association with chemosensitivity (Fisher exact test, *p >* 0.5).

**Fig 2:**
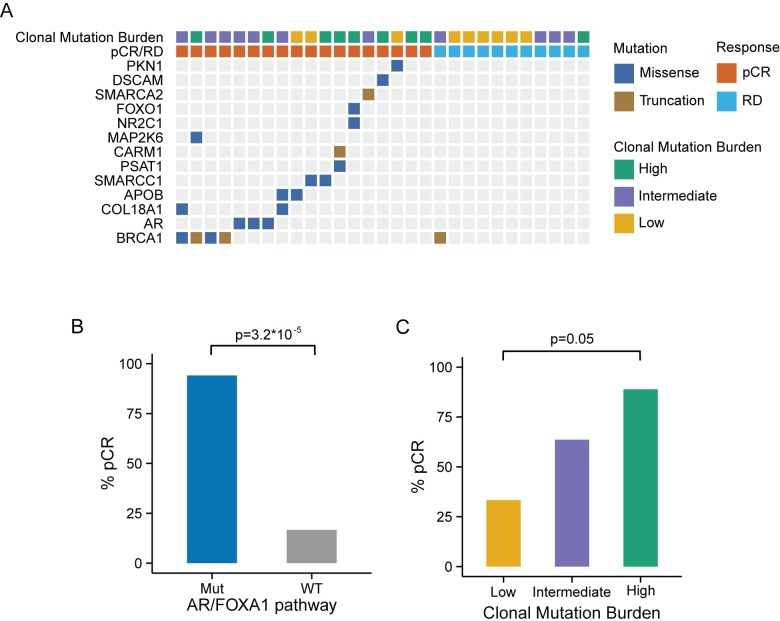
Genomic markers predictive of chemosensitivity in the MDACC triple negative breast cancer dataset. (A) Mutations in combined “regulation of androgen receptor activity” and “FOXA1 transcription factor network” pathways in the MDACC samples. (B) pCR rate by mutation status of the AR/FOXA1 pathway. The pCR rate was significantly higher (94.1%) in the cancers carrying at least one mutation in the AR/FOXA1 pathway compared to WT (16.6%) by the Fisher exact test (*p* < 0.001). (C) pCR rate by CMB category. CMB was defined based on MR and clonal heterogeneity. Tumors with high CMB appear to be extremely chemosensitive. AR, androgen receptor; CMB, clonal mutation burden; MDACC, MD Anderson Cancer Center; MR, mutation rate; Mut, mutation; pCR, pathologic complete response; RD, residual disease; WT, wild-type.

Functional mutations in nine canonical biological pathways were associated with chemotherapy response (Fisher exact test, *p <* 0.05; S1 Table). Due to the small size of the response groups, we applied a bootstrap strategy to evaluate the robustness of these associations under resampling (S2 Fig), and a permutation strategy to assess their significance (S1 Table). The top two pathways, “regulation of androgen receptor activity” and “FOXA1 transcription factor network,” remained significant, with mutations in both pathways being associated with pCR. Considering their substantial overlap (14 genes in common out of 59 in the androgen receptor [AR] pathway and 58 in the FOXA1 pathway), we merged the two pathways into the “AR- and FOXA1-regulated network.” Tumors carrying mutations in the AR/FOXA1 pathway had a significantly higher pCR rate (94.1% compared to 16.6% in tumors without such mutations, *q* = 0.02 after Bonferroni correction; Fig 2B). Furthermore, functional mutations occurred almost exclusively in chemosensitive tumors (21/22, or 95% of mutations) except for a single truncating *BRCA1* mutation found in one RD tumor with AJCC stage IIIB cancer but minimal residual cancer burden (Fig 2A; Table 1, sample id 757_004_004). At the gene level, 13 genes had at least one mutation affecting 17 chemosensitive patients (58.6%) across the entire cohort (Fig 2A). In most cases, different genes were mutated in individual tumors, and despite the strong association observed at the pathway level, none of these genes individually had a significant association with pCR. Among them, *BRCA1* was the most frequently mutated gene (17%), and four of the mutations observed in the pCR cohort were associated with hereditary breast cancers in the ClinVar database (S3 Fig). The *BRCA1* mutation found in the tumor with RD (Fig 2A; see also S3 Fig) is a stop-gain mutation also reported in ClinVar as pathogenic, but due to lack of RNAseq data we could not assess the relative expression of mutant and WT *BRCA1* transcripts.

### Mutational Burden and Clonality as Predictors of Chemosensitivity in Triple Negative Breast Cancer

To assess whether broad genomic measures that capture the overall burden and heterogeneity of somatic mutations are predictive of chemosensitivity, we calculated the MR and MATH score in each TNBC tumor. We also estimated tumor clonality by applying clonal decomposition to the somatic mutation profile of each tumor, and confirmed a positive correlation between the MATH score and the estimated number of clones in each tumor (S4 Fig). We sought to combine MR and MATH into a composite score, which we called CMB, that captures both the clonality of a tumor and the number of somatic mutations per clone. MR and MATH scores were median-dichotomized in the cohort, and CMB categories were defined as low (low MR, high MATH), high (high MR, low MATH), or intermediate (all others). There was an increasing tendency in the average number of mutations per clone from low to high CMB (S5 Fig). The pCR rate in the low, intermediate, and high CMB tumors was 33%, 64%, and 89%, respectively (Fig 2C), suggesting that tumors with a high number of mutations per clone (small number of clones, high MR) have significantly better response (*p* = 0.05) than low CMB tumors, which may be subclonal or have an overall low MR. The CMB categories were not significantly associated with other clinical or pathologic characteristics such as tumor stage or grade, or with patient’s age.

### AR/FOXA1 Mutations Predict Better Clinical Outcome in Triple Negative Breast Cancer Tumors from TCGA

To validate our findings, we selected TCGA TNBC samples based on IHC status and PAM50 subtype, excluded stage IV cancers and cases with short follow-up, and downloaded available processed data of WES, RNAseq, DNA methylation, and copy number variation (Fig 1; S2 Table).

**Fig 3:**
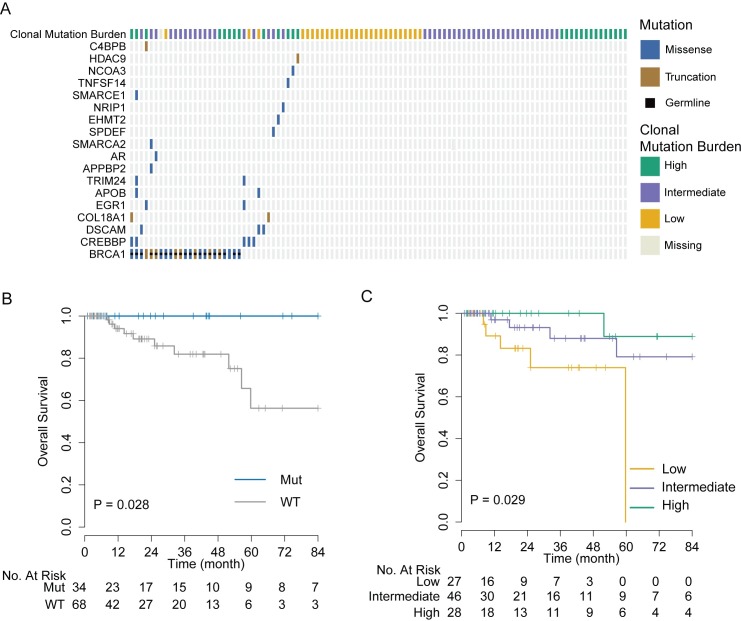
Validation of genomic markers of chemosensitivity in the TCGA triple negative breast cancer cohort. (A) Somatic and germline mutations in AR/FOXA1 pathway and CMB in the TCGA TNBC cohort. (B) Kaplan-Meier estimator of overall survival outcomes by AR/FOXA1 mutational status. Tumors carrying at least one mutation in the AR/FOXA1 pathway had significantly better overall survival compared to WT tumors (log-rank test, *p* = 0.028). (C) Overall survival outcomes of TCGA TNBC cases by CMB category. Cases with high CMB have significantly better overall survival (log-rank test, *p* = 0.025). AR, androgen receptor; CMB, clonal mutation burden; TCGA, The Cancer Genome Atlas; TNBC, triple negative breast cancer; WT, wild-type.

Among the 102 TNBC cases from the TCGA cohort with available exome sequencing data, 19 had at least one functional somatic mutation in the AR/FOXA1 pathway and 35 had at least one functional somatic mutation in the AR/FOXA1 pathway or at least one germline *BRCA1* mutation. As with the MDACC cohort, *BRCA1* was most frequently mutated among this gene set, with 21 (20%) patients carrying germline mutations and two (2%) carrying somatic mutations (Fig 3A). Patients with at least one mutation in one of the genes in this pathway had excellent overall survival, while those with WT genes had significantly worse outcomes (*p* = 0.028; Fig 3B). We observed a similar trend when we considered only somatic mutations (S6 Fig).

### Validation of Clonal Mutation Burden as Biomarker of Chemosensitivity in TCGA

We computed the MR and MATH score in 101 TCGA TNBC cases with available exome sequence alignment data. Also in this cohort, the MATH score was positively correlated with the estimated number of clones (*R* = 0.44, *p* < 0.001; S7 Fig). Cases were stratified into three categories of CMB using the same criteria as with the MDACC cohort. Tumors with high CMB harbored a significantly higher average number of mutations per clone than tumors with low CMB (S8 Fig), and, consistent with the MDACC cohort, patients with these tumors had a significantly improved overall survival rate (*p* = 0.029) compared to patients with tumors with low CMB (Fig 3C).

### BRCA-Deficient Triple Negative Breast Cancer Tumors Defined by Low Wild-Type BRCA1/2 Expression

*BRCA1* was the most frequently mutated gene among the gene set that we found to be associated with chemosensitivity: it was mutated in about 20% of TNBC cases in both cohorts. Loss of function variants in *BRCA1* or *BRCA2* genes lead to homologous recombination defects and may contribute to sensitivity to platinum-based chemotherapy, but it is yet unclear whether this deficiency is associated with improved benefit from standard-of-care ACT chemotherapy. Besides germline and somatic mutations, deletions or epigenetic silencing can also result in DNA repair deficiency in a large proportion of TNBC cases. Here we systematically evaluated BRCA deficiency in TNBC using integrated analysis of DNA sequencing, DNA methylation, and RNAseq data from TCGA and assessed its association with clinical outcome.

**Fig 4:**
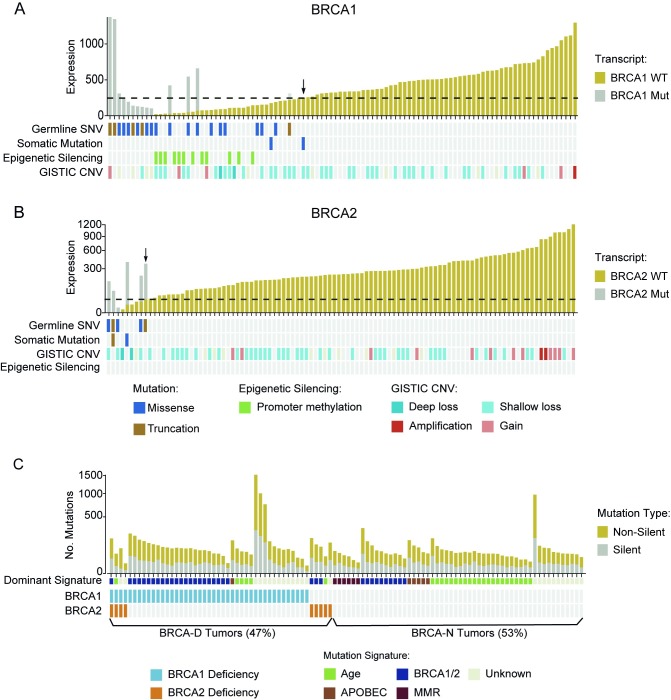
BRCA deficiency characterized by low wild-type transcript abundance defines a unique subtype of triple negative breast cancer. (A) Low WT BRCA1 abundance summarizes BRCA1 deficiency through multiple types of genomic aberrations. Arrow indicates the BRCA1 deficiency threshold. (B) Low WT BRCA2 abundance summarizes BRCA2 inactivation by multiple types of genomic aberrations. Arrow indicates BRCA2 deficiency threshold. (C) Number of silent or non-silent mutations, mutation signatures, and BRCA deficiency in TCGA TNBCs. BRCA-D, BRCA-deficient; BRCA-N, BRCA-normal; CNV, copy number variation; MMR, mismatch repair; Mut, mutation; SNV, single nucleotide variant; TCGA, The Cancer Genome Atlas; TNBC, triple negative breast cancer; WT, wild-type.

Among the 101 TNBCs from the TCGA dataset, 21 tumors (20.5%) had inactivating germline SNP or somatic mutations in *BRCA1*, four (3.9%) in *BRCA2*, and two (1.9%) in both (Fig 4A and 4B). One hotspot germline SNP (rs1799950; Q356R in exon 10) was particularly common in this cohort, appearing in 15 cases (S3 Fig). Furthermore, we assessed the copy number variation in *BRCA1/2* and promoter hypermethylation in *BRCA1*. Although different types of abnormalities are associated with *BRCA1/2* inactivation, they all result in low expression of functional WT BRCA transcript. In mutation carriers, WT transcript abundance was determined from the overall expression, and MAF from RNAseq data. As expected, the abundance of WT BRCA1/2 transcripts was significantly lower in mutation carriers than in non-carriers (*p <* 10^−16^; Fig 4A and 4B). We therefore defined the BRCA1/2 deficiency threshold as the maximum level of WT BRCA1/2 transcript expressed in BRCA1/2 mutation carriers. That threshold is indicated by an arrow in Fig 4A and 4B. Tumors with WT BRCA1/2 transcript abundance below the corresponding threshold were classified as BRCA-D. This includes all the cases with germline or somatic mutations, or deep loss, or epigenetic silencing of the BRCA genes.

Based on this expanded definition of BRCA deficiency, 48 TNBC cases (47%) were characterized as BRCA-D. Specifically, 43 cases (42%) were BRCA1 deficient, nine (9%) were BRCA2 deficient, and four (4%) were deficient in both (Fig 4C). This definition captured all the cases with BRCA mutation, BRCA1 promoter methylation, or BRCA deletion, but also an additional nine cases (9%) that expressed low levels of WT BRCA transcripts for unknown reasons. We applied non-negative linear regression to 76 tumors with available mutation context data to estimate the proportion of mutations explained by the BRCA, age, APOBEC, and mismatch repair mutational signatures in each sample. The BRCA signature was dominant in 78.8% BRCA-D tumors (*p* < 0.001), the age signature was dominant in BRCA-N tumors (*p* = 0.004), and the APOBEC signature was more often present in BRCA-N tumors (14%) than in BRCA-D tumors (*p* = 0.03; Fig 4C). Furthermore, BRCA-D tumors were associated with significantly higher MRs based on non-silent (*p* < 0.001) or silent (*p* = 0.02) mutations, but had clonal heterogeneity (*p* = 0.55) similar to that of BRCA-N TNBC tumors (S9 Fig).

### Triple Negative Breast Cancer Tumors of BRCA-Deficient Subtype Benefit from Anthracycline/Taxane Chemotherapy

**Fig 5:**
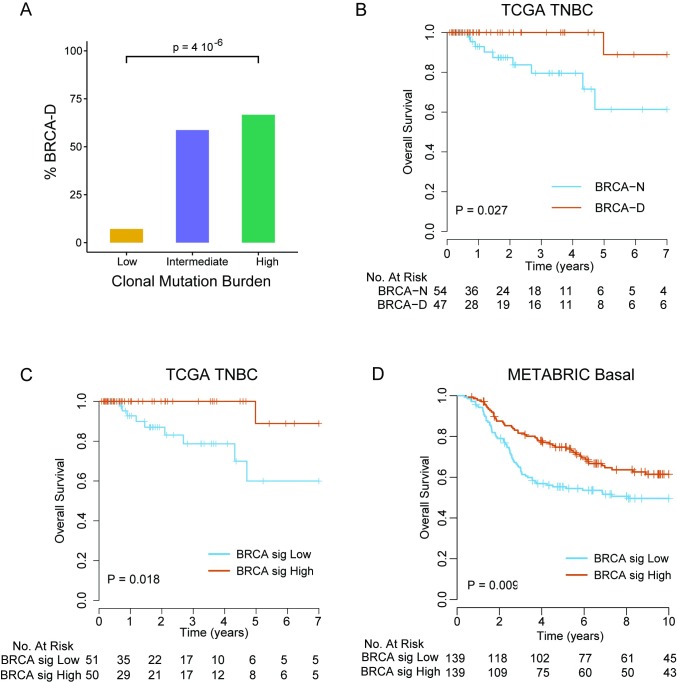
BRCA-deficient subtype signature identifies triple negative breast cancer patients with improved survival with anthracycline/taxane chemotherapy. (A) Proportion of BRCA-D tumors among TNBC tumors in different CMB categories. (B) Overall survival of TNBC patients by BRCA deficiency status in the TCGA cohort. (C) Overall survival of TNBC patients in the TCGA cohort by predicted BRCA deficiency status using the developed gene signature (high corresponds to BRCA-D; low to BRCA-N). (D) Overall survival of TNBC patients in the METABRIC cohort by predicted BRCA deficiency status using the developed gene signature (high corresponds to BRCA-D; low to BRCA-N). *p*-Values are from the log-rank test. BRCA-D, BRCA-deficient; BRCA-N, BRCA-normal; CMB, clonal mutation burden; TCGA, The Cancer Genome Atlas; TNBC, triple negative breast cancer.

We found that expression of WT BRCA transcripts defines a new TNBC subtype (BRCA-D) characterized by high MR but typical clonal heterogeneity. Moreover, TNBC tumors with high CMB were highly enriched in BRCA-D tumors (67%) compared to tumors of low CMB (7%) (*p* < 0.001; Fig 5A), suggesting that BRCA1/2-mediated homologous recombination deficiency is associated with tumors of high CMB that were extremely sensitive to ACT chemotherapy in the MDACC cohort (Fig 2C).

Patients with BRCA-D tumors had 100% 4-y overall survival, compared to 79.5% (95% CI 66.6%–94.9%) for BRCA-N tumors (log-rank test, *p* = 0.018; Fig 5B), in the TCGA TNBC cohort. To further validate this finding in an independent dataset of TNBC cases without requiring RNA and DNA sequencing data, we developed a gene expression signature that predicts BRCA-D status in the TCGA dataset. We identified 24 genes that were strongly overexpressed and 26 genes that were underexpressed in BRCA-D compared to BRCA-N cases (arbitrary cutoff, unadjusted *p <* 0.002; S10 Fig). Among the 50 genes identified (S3 Table), *BRCA1* was the gene most strongly associated with BRCA deficiency (*p* < 0.001). For each sample, a BRCA deficiency signature score was computed as the mean expression of the 24 overexpressed genes minus the mean expression of the 26 underexpressed genes. The score was median-dichotomized to predict BRCA-D status if high or BRCA-N status if low. The predictor of BRCA deficiency status had a sensitivity of 85.4% and specificity of 81.5% for predicting BRCA deficiency status in the TCGA cohort where it was developed. Furthermore, the patients with predicted BRCA-D tumors had significantly better overall survival than patients with predicted BRCA-N tumors (log-rank test, *p* = 0.013; Fig 5C). We applied the BRCA deficiency signature to an independent cohort of 278 chemotherapy-treated TNBC cases from the METABRIC cohort, which validated that patients with tumors predicted to be BRCA-D by the gene signature had significantly better overall survival compared to patients with tumors predicted to be BRCA-N (log-rank test, *p* = 0.009; Fig 5D).

### Link between Clonal Mutation Burden, BRCA Deficiency, and Immune Activation in Triple Negative Breast Cancer

**Fig 6:**
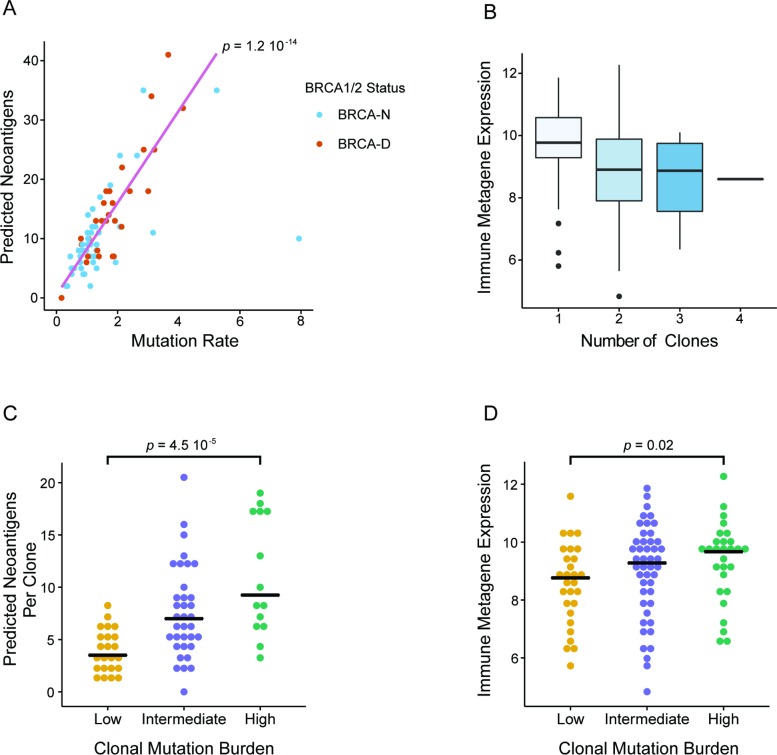
Clonal mutation burden and BRCA deficiency linked to immune activation in triple negative breast cancer. (A) Correlation between overall mutation rate and number of predicted neoantigens in TNBC. (B) Expression of immune metagenes as a function of the estimated clonality of tumors. Clonal tumors are associated with a significantly enhanced immune presence. (C) Estimated number of predicted neoantigens per clone in TNBC cases of different CMB categories. (D) Expression of immune metagenes in TNBC cases with different CMB. Both the number of neoantigens per clone and the immune presence are greater in tumors with high CMB. BRCA-D, BRCA-deficient; BRCA-N, BRCA-normal; CMB, clonal mutation burden; TCGA, The Cancer Genome Atlas; TNBC, triple negative breast cancer.

In our analysis we discovered that BRCA deficiency is associated with higher CMB and that both BRCA deficiency and high CMB are predictive of chemosensitivity in TNBC. Given that a high prevalence of TILs has often been associated with better response in TNBC, we wanted to further investigate whether high CMB and BRCA deficiency are associated with higher immune activation. In the TCGA TNBC cohort, we observed a strong correlation between the overall MR and the number of predicted neoantigens (Spearman correlation = 0.76; Fig 6A). Due to higher overall MR, BRCA-D tumors had a significantly greater number of predicted neoantigens compared to BRCA-N tumors (*p* = 0.003; S11 Fig). Additionally, to estimate immune cell prevalence we used the average expression of the lymphocyte-specific genes *GZMB*, *PRF1*, *CXCL13*, *IRF1*, *IKZF1*, and *HLA-E* in each tumor sample [39]. Interestingly, tumors with predominantly clonal mutations were associated with higher immune presence compared to those with subclonal mutations (*p* = 0.003; Fig 6B), implying a negative association between clonal heterogeneity and immune response (S12 Fig). Therefore, tumors with high CMB harbor a greater number of predicted neoantigens per clone (Fig 6C), which elicit a higher immune response (Fig 6D). In summary, our analysis suggests a connection between BRCA deficiency status and high CMB, which results in a greater number of clonal neoantigens, leading to immune activation and potentially mediating enhanced response to ACT chemotherapy in TNBC.

## Discussion

We report results from an integrated genomic analysis of a TNBC cohort deliberately selected to represent extremely chemosensitive tumors and tumors highly resistant to standard-of-care ACT chemotherapy. Although no significant associations were identified between recurrent functional somatic mutations in specific genes and chemotherapy response, aggregating at the pathway level revealed that mutations occurring in two pathways, “regulation of androgen receptor activity” and “FOXA1 transcription factor network,” were significantly associated with pCR in TNBC (94% pCR rate in tumors with mutated pathways versus 17% in tumors without such mutations). Furthermore, TNBC patients from the TCGA cohort whose tumors had at least one mutation in the above pathways had excellent survival, with no deaths observed in 4 y when treated with ACT-containing regimens.

TNBC is highly heterogeneous, and up to six different subtypes have been recognized by transcriptional profiling, each associated with different clinical outcomes and responses to therapy [40,41]. One subtype, the luminal androgen receptor (LAR) subtype, is characterized by luminal gene expression driven by the AR and is generally associated with low response to chemotherapy [40]. The AR is expressed in about 10%–40% of TNBCs, but its role in prognosis or as a potential therapeutic target in TNBC has remained controversial [42]. In TNBC, signaling through the AR is hypothesized to mimic ER signaling, initiating transcriptional activation that promotes cell growth through the involvement of the transcription factor FOXA1 [43]. This has provided a justification for targeting AR in AR-positive TNBC. Recent single-arm phase II studies that evaluated the effect of the AR antagonists bicalutamide and enzalutamide in metastatic AR+ TNBC reported 6-mo clinical benefit rates of 19% and 29%, respectively [44,45], suggesting a direct role of AR in this TNBC subtype. Our results are consistent with the above observations, suggesting that mutations in the AR/FOXA1 pathway could result in abrogation of AR-related signaling, resulting in improved sensitivity to standard chemotherapy and better overall survival.

TNBC tumors are characterized by broad genomic and transcriptional heterogeneity [10,14]. The extent of genomic and transcriptional heterogeneity in tumors appears to be associated with resistance to chemotherapy in TNBC [10] and with worse prognosis in head and neck cancer [30]. Furthermore, high somatic mutation load has been linked to favorable outcomes in pancreatic cancer [46] but to worse prognosis in ER-positive breast cancer [47]. In our assessment of broad genomic measures as potential predictors of chemosensitivity, we found that patients with tumors with high CMB, defined as high mutational load but low clonality or a high number of somatic mutations per clone, have a significantly higher pCR rate (89%) and excellent survival (no deaths in 4 y in the TCGA cohort) compared to patients with tumors with low CMB (pCR rate of 33%). Therefore, the clonality of a tumor appears be critical in determining chemotherapy sensitivity and survival outcome. Tumors with high mutation load, for instance due to defective DNA damage response pathways, are sensitive to chemotherapy provided that they are not subclonal, that is, they do not contain mutations of lower variant allele frequencies that would have originated from subclones arising later in the tumor’s clonal expansion. Subclonal tumors contain not only the clonal mutations that were present in the founding cell but also subclonal mutations that emerged in subsequent clones during clonal expansion and thus exhibit broader genetic heterogeneity, which contributes to resistance to chemotherapy [48–50].

BRCA1 and BRCA2 are critical for the process of DNA repair by homologous recombination repair (HRR), and deficient HRR makes cancers more susceptible to DNA-damaging agents. Familial *BRCA1* or *BRCA2* mutant breast tumors tend to have a TNBC phenotype and often exhibit extreme levels of genomic instability [51]. The “BRCAness” phenotype is more broadly defined as defective HRR, driven not only by germline *BRCA1* and *BRCA2* mutations but also by somatic mutations or other alterations in these or other genes involved in HRR [52]. Indeed, ovarian tumors with *BRCA1* or *BRCA2* mutations, either germline or somatic, are associated with higher mutational burden and better survival outcomes following treatment with platinum-based chemotherapy [53] or PARP inhibitors [54]. In TNBC, germline *BRCA1* mutation carriers were found to have higher pCR rates to neoadjuvant ACT chemotherapy compared to non-carriers (46% versus 22%) and significantly better survival outcomes [55]. Similar results were reported in TNBC with promoter methylation of *BRCA1*, where *BRCA1* methylation was associated with better survival outcomes following adjuvant ACT chemotherapy [56]. Our broad definition of BRCA deficiency from RNAseq data based on the level of WT BRCA transcripts incorporates the effect of all genomic aberrations leading to inadequate levels of functional BRCA. This broader definition of BRCA deficiency included 46% of the TNBC tumors in the TCGA cohort; this subset had an excellent survival outcome (100% 4-y survival) following adjuvant ACT chemotherapy. Although the evidence provided by the survival data in the TCGA cohort is somewhat limited due to shorter follow-up and a lower number of deaths observed than expected for TNBC, the effect of BRCA deficiency was confirmed in the METABRIC cohort, which has 10 y of follow-up and more representative overall survival for TNBC. Our analysis therefore suggests that a definition of BRCA deficiency based on RNAseq could be a clinically useful biomarker of chemosensitivity for TNBC.

Immunotherapy is now emerging as a potentially viable therapeutic option for TNBC patients [57], but this treatment is expected to be effective only for a subset of the patients. Antibodies against programmed death 1 (PD-1) were significantly more effective in mismatch-repair-deficient colorectal cancers, most likely due to an increased number of neoantigens in these tumors [31]. We observed that BRCA-D TNBC tumors are characterized by high CMB and carry a greater number of predicted neoantigens that tend to be clonal. This could be the reason for the higher level of immune infiltration observed in these tumors [58] and may have contributed to the improved response to ACT chemotherapy that we observed. These results suggest that the combination of immunotherapies with ACT chemotherapy or PARP inhibitors might be an effective strategy for treating BRCA-D tumors. This is currently being evaluated in a phase I/II study in BRCA-D ovarian cancer (ClinicalTrials.gov; study ID NCT02571725).

In summary, we have provided an integrated characterization of the chemotherapy response phenotypes in TNBC. The strong connection of ACT chemosensitivity and immune activity with a new transcriptionally defined BRCA-D phenotype could help inform future therapeutic strategies for TNBC patients.

Limitations of our single-institution retrospective study include the small size of the discovery cohort and potential selection bias as samples were included based on both chemotherapy response and availability of residual biopsy materials for DNA isolation. Given the genomic heterogeneity of TNBC, this might limit the generalizability of our results. Another limitation is the lack of tumor-matched normal DNA for these tumors, which may result in reduced sensitivity and specificity for detecting somatic mutations in this cohort. Although our observation that mutations in AR/FOXA1 genes are associated with better outcomes in ACT-treated TNBC patients was validated in the TCGA cohort, the low number of events observed in this cohort limits the power of the analysis. Yet, our key finding that BRCA-D TNBC tumors identified by the BRCA deficiency signature are indeed associated with better outcomes after chemotherapy was confirmed in both the TCGA and METABRIC datasets. Although these findings will require validation in larger multi-institutional datasets, preferably originating from prospective clinical studies, they could provide the impetus for examining BRCA deficiency in TNBC in the context of increased CMB, with potentially improved response to immunotherapies.

## Supporting Information

S1 STROBE Checklist STROBE checklist. (PDF)

S1 Fig Non-silent mutations of *TP53* in patients from the MDACC cohort with pathologic complete response or extensive residual disease. (TIFF)

S2 Fig Bootstrap evaluation of the significance of the association between mutational status of pathways and chemotherapy response. The frequency of occurrence of significant associations (by Fisher exact test *p <* 0.05) in 100 bootstrap resampling iterations of the mutation matrix is plotted against the *p*-value obtained from the original MDACC cohort for each pathway. To assess the effect of sampling variation, we resampled the patient cohort with replacement to a final bootstrapped cohort with a different number of responder (pCR) and non-responder (RD) cases, as indicated in the figure (all mutations for each selected patient were included).
(TIFF)

S3 Fig Non-silent mutations of *BRCA1* in patients from the MDACC cohort with pathologic complete response or extensive residual disease. Also shown are the mutations from the TGCA TNBC cohort.
(TIFF)

S4 Fig Relation between MATH score and the estimated total number of clones in the MDACC triple negative breast cancer cohort. (TIFF)

S5 Fig Estimated average number of mutations per clone in groups of different clonal mutation burden in the MDACC triple negative breast cancer cohort. (TIFF)

S6 Fig Overall survival outcomes in TCGA triple negative breast cancer cohort by mutational status of genes in the AR/FOXA1 pathway when only the somatic mutations are considered. (TIF)

S7 Fig Relation between MATH score and the estimated total number of clones in the TCGA triple negative breast cancer cohort. (TIFF)

S8 Fig Estimated average number of mutations per clone in groups of different clonal mutation burden in the TCGA triple negative breast cancer cohort. (TIFF)

S9 Fig Comparison of mutation rate of non-silent mutations and silent mutations and MATH score between BRCA-deficient and BRCA-normal triple negative breast cancer tumors from the TCGA cohort. (TIFF)

S10 Fig Heatmap of the BRCA deficiency discriminant gene expression profiles in the TCGA triple negative breast cancer cohort. The samples are ordered by BRCA deficiency status, and the genes are ordered by hierarchical clustering. (TIFF)

S11 Fig Number of predicted neoantigens in BRCA-deficient and BRCA-normal triple negative breast cancer tumors from the TCGA cohort. (TIFF)

S12 Fig Negative association between expression of the immune metagene and clonal heterogeneity as captured by the MATH score in the triple negative breast cancer cases from the TCGA cohort. (TIFF)

S1 Table Nine pathways that achieved nominal significance to predict chemotherapy response in the MDACC cohort. (XLSX)

S2 Table Data availability from different platforms in the TCGA triple negative breast cancer cohort. (XLSX)

S3 Table Fifty genes included in the BRCA deficiency signature. (XLSX)

## Abbreviations

ACT: anthracycline/taxane
AR: androgen receptor
BRCA-D: BRCA-deficient
BRCA-N: BRCA-normal
CMB: clonal mutation burden
ER: estrogen receptor
HRR: homologous recombination repair
IHC: immunohistochemistry
INDEL: insertion/deletion
MAF: mutant allele frequency
MATH: mutant allele tumor heterogeneity
MDACC: MD Anderson Cancer Center
MR: mutation rate
PARP: poly(ADP-ribose) polymerase
pCR: pathologic complete response
PR: progesterone receptor
RD: residual disease
RNAseq: RNA sequencing
TCGA: The Cancer Genome Atlas
TIL: tumor-infiltrating lymphocyte
TNBC: triple negative breast cancer
WES: whole exome sequencing
WT: wild-type
