# Supplementary material for: Predictors of Chemosensitivity in Triple Negative Breast Cancer: An Integrated Genomic Analysis
Source: PLoS Med. 2016 Dec 13;13(12):e1002193. doi: 10.1371/journal.pmed.1002193 (PMC5154510; doi:10.1371/journal.pmed.1002193)
Supplement: S1 STROBE Checklist — (PDF) [file pmed.1002193.s001.pdf]

STROBE Statement—Checklist of items that should be included in reports of ***cohort studies***

	Item No	Recommendation	Reference in Manuscript
**Title and abstract**	1	(*a*) Indicate the study's design with a commonly used term in the title or the abstract	An integrated genomic analysis...
		(*b*) Provide in the abstract an informative and balanced summary of what was done and what was found	Structured abstract Author summary
**Introduction**			
Background/rationale	2	Explain the scientific background and rationale for the investigation being reported	Introduction, paragraphs 1-3
Objectives	3	State specific objectives, including any prespecified hypotheses	Introduction, paragraph 4
**Methods**			
Study design	4	Present key elements of study design early in the paper	Introduction/paragraph 4, Methods/sample collection and datasets
Setting	5	Describe the setting, locations, and relevant dates, including periods of recruitment, exposure, follow-up, and data collection	Methods, MDACC cohort
Participants	6	(*a*) Give the eligibility criteria, and the sources and methods of selection of participants. Describe methods of follow-up	Described in previous study where the samples were collected; reference provided on p 8
		(*b*) For matched studies, give matching criteria and number of exposed and unexposed	Criteria for selecting TNBC cases from other cohorts provided (Fig. 1, p. 8-9)
Variables	7	Clearly define all outcomes, exposures, predictors, potential confounders, and effect modifiers. Give diagnostic criteria, if applicable	Outcomes clearly defined: pCR for neoadjuvant, Overall Survival for adjuvant
Data sources/ measurement	8*	For each variable of interest, give sources of data and details of methods of assessment (measurement). Describe comparability of assessment methods if there is more than one group	Details for sequencing and analysis provided in methods
Bias	9	Describe any efforts to address potential sources of bias	Validation in independent cohorts (TCGA and METABRIC)
Study size	10	Explain how the study size was arrived at	Explained on p.8 under MDACC cohort
Quantitative variables	11	Explain how quantitative variables were handled in the analyses. If applicable, describe which groupings were chosen and why	Explained throughout (clonal mutation burden, BRCA-deficient status)
Statistical methods	12	(*a*) Describe all statistical methods, including those used to control for confounding	Described in methods
		(*b*) Describe any methods used to examine subgroups and	Described methods for

		interactions	assessing association between BRCA-D status and OS
		(*c*) Explain how missing data were addressed	Only cases with complete data for the given analysis were used
		(*d*) If applicable, explain how loss to follow-up was addressed	Standard censoring in survival analysis
		(*e*) Describe any sensitivity analyses	None done
**Results**			
Participants	13*	(a) Report numbers of individuals at each stage of study—eg numbers potentially eligible, examined for eligibility, confirmed eligible, included in the study, completing follow-up, and analysed	P. 8 and Fig. 1
		(b) Give reasons for non-participation at each stage	P. 8 and Fig. 1
		(c) Consider use of a flow diagram	Fig 1 for TCGA cohgort
Descriptive data	14*	(a) Give characteristics of study participants (eg demographic, clinical, social) and information on exposures and potential confounders	S1 Table
		(b) Indicate number of participants with missing data for each variable of interest	S3 Table
		(c) Summarise follow-up time (eg, average and total amount)	For TCGA and METABRIC summarized in original publications
Outcome data	15*	Report numbers of outcome events or summary measures over time	Figs 3, 4
Main results	16	(*a*) Give unadjusted estimates and, if applicable, confounder-adjusted estimates and their precision (eg, 95% confidence interval). Make clear which confounders were adjusted for and why they were included	Unadjusted and adjusted p-values for pathway analysis in S2 Table
		(*b*) Report category boundaries when continuous variables were categorized	P 19 for BRCA-D signature
		(*c*) If relevant, consider translating estimates of relative risk into absolute risk for a meaningful time period	
Other analyses	17	Report other analyses done—eg analyses of subgroups and interactions, and sensitivity analyses	Reported in results (BRCA-D analysis)
**Discussion**			
Key results	18	Summarise key results with reference to study objectives	Throughout discussion
Limitations	19	Discuss limitations of the study, taking into account sources of potential bias or imprecision. Discuss both direction and magnitude of any potential bias	End of discussion
Interpretation	20	Give a cautious overall interpretation of results considering objectives, limitations, multiplicity of analyses, results from similar studies, and other relevant evidence	Throughout discussion
Generalisability	21	Discuss the generalisability (external validity) of the study	Need for additional

		results	validation discussed
**Other information**			
Funding	22	Give the source of funding and the role of the funders for the present study and, if applicable, for the original study on which the present article is based	Provided in Acknowledgments

*Give information separately for exposed and unexposed groups.

Note: An Explanation and Elaboration article discusses each checklist item and gives methodological background and published examples of transparent reporting. The STROBE checklist is best used in conjunction with this article (freely available on the Web sites of PLoS Medicine at http://www.plosmedicine.org/, Annals of Internal Medicine at http://www.annals.org/, and Epidemiology at http://www.epidem.com/). Information on the STROBE Initiative is available at http://www.strobe-statement.org.
