# Supplementary figures and images for: Predictors of Chemosensitivity in Triple Negative Breast Cancer: An Integrated Genomic Analysis
Source: PLoS Med. 2016 Dec 13;13(12):e1002193. doi: 10.1371/journal.pmed.1002193 (PMC5154510; doi:10.1371/journal.pmed.1002193)

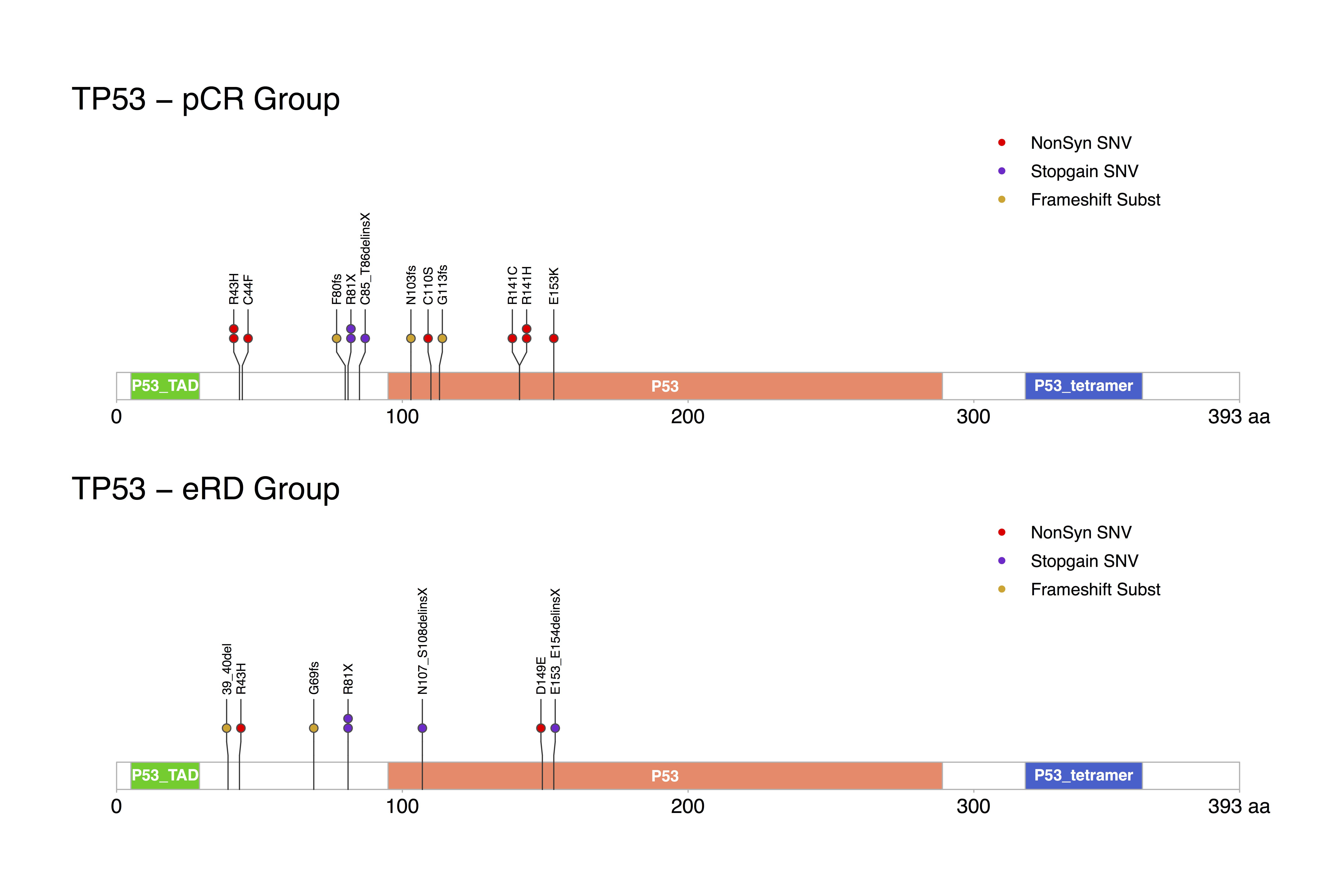

Supplement: S1 Fig — (TIFF) [file pmed.1002193.s002.tiff]

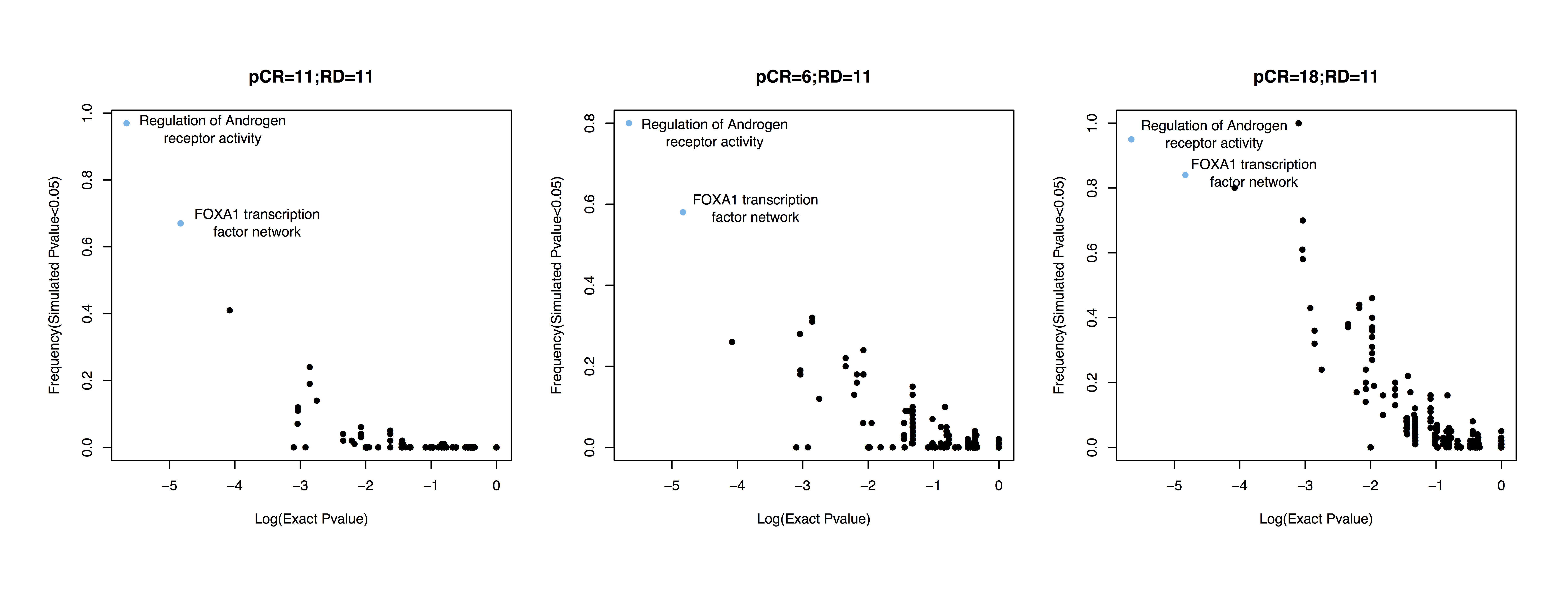

Supplement: S2 Fig — The frequency of occurrence of significant associations (by Fisher exact test p < 0.05) in 100 bootstrap resampling iterations of the mutation matrix is plotted against the p-value obtained from the original MDACC cohort for each pathway. To assess the effect of sampling variation, we resampled the patient cohort with replacement to a final bootstrapped cohort with a different number of responder (pCR) and non-responder (RD) cases, as indicated in the figure (all mutations for each selected patient were included). (TIFF) [file pmed.1002193.s003.tiff]

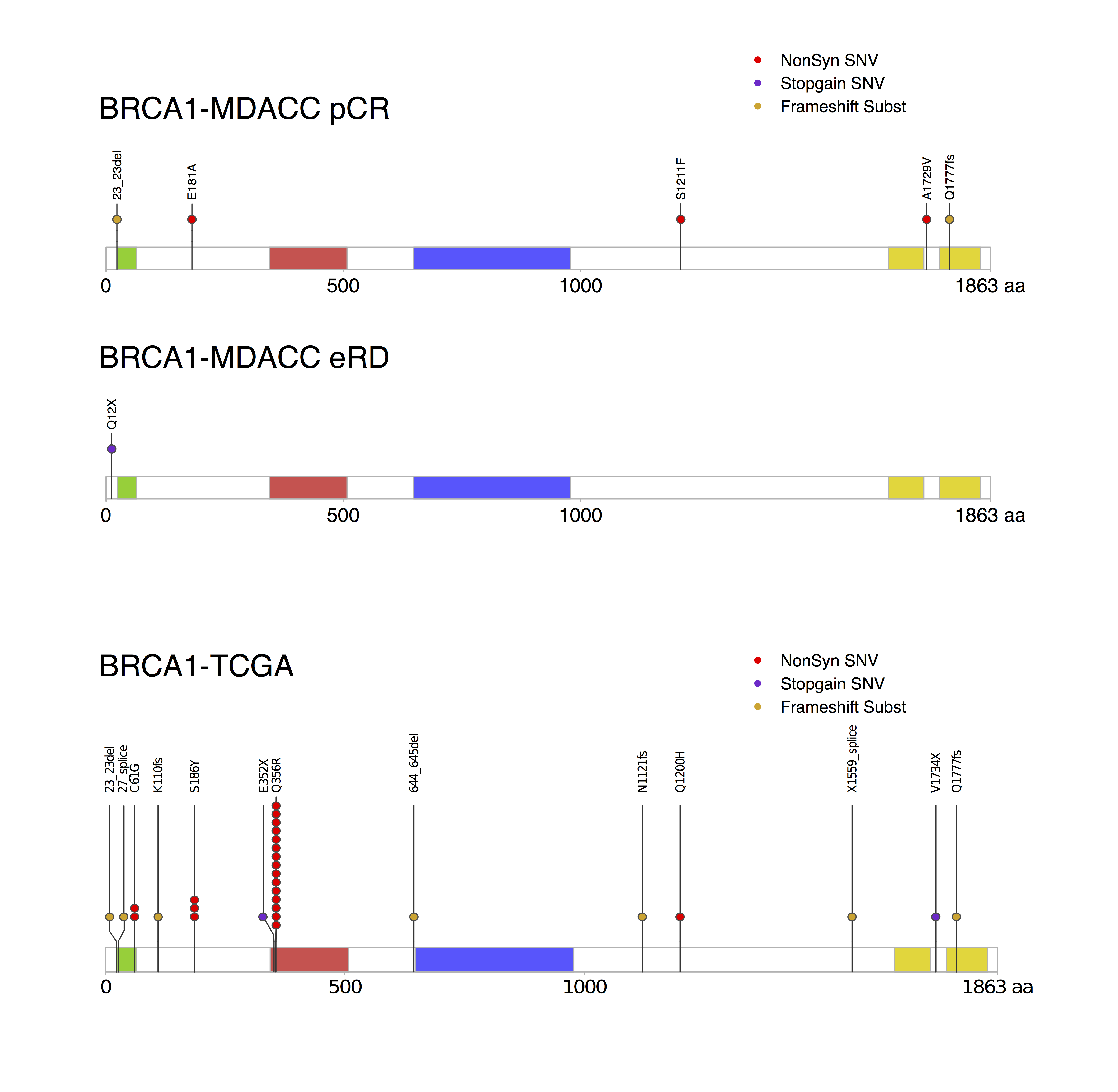

Supplement: S3 Fig — Also shown are the mutations from the TGCA TNBC cohort. (TIFF) [file pmed.1002193.s004.tiff]

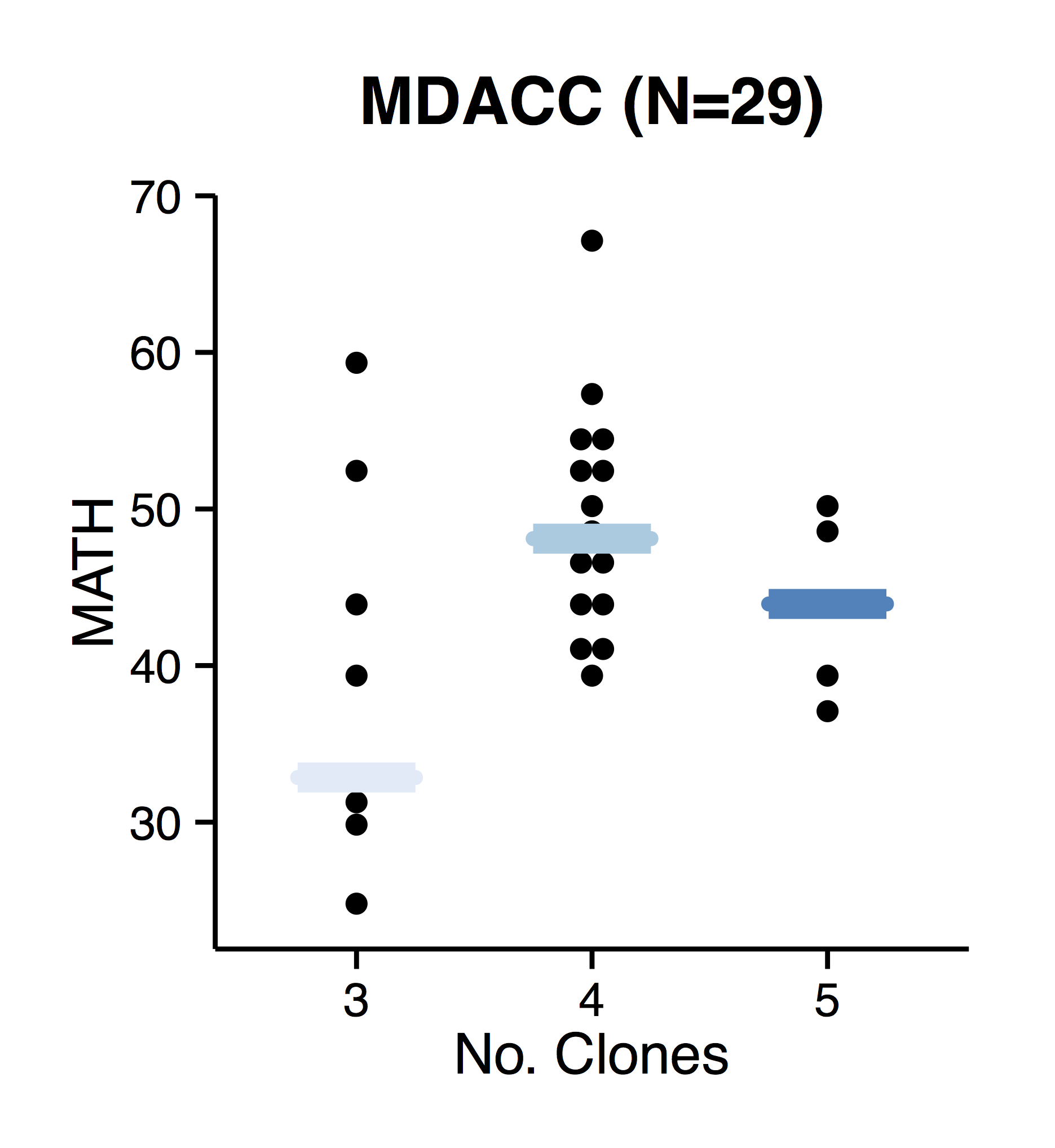

Supplement: S4 Fig — (TIFF) [file pmed.1002193.s005.tiff]

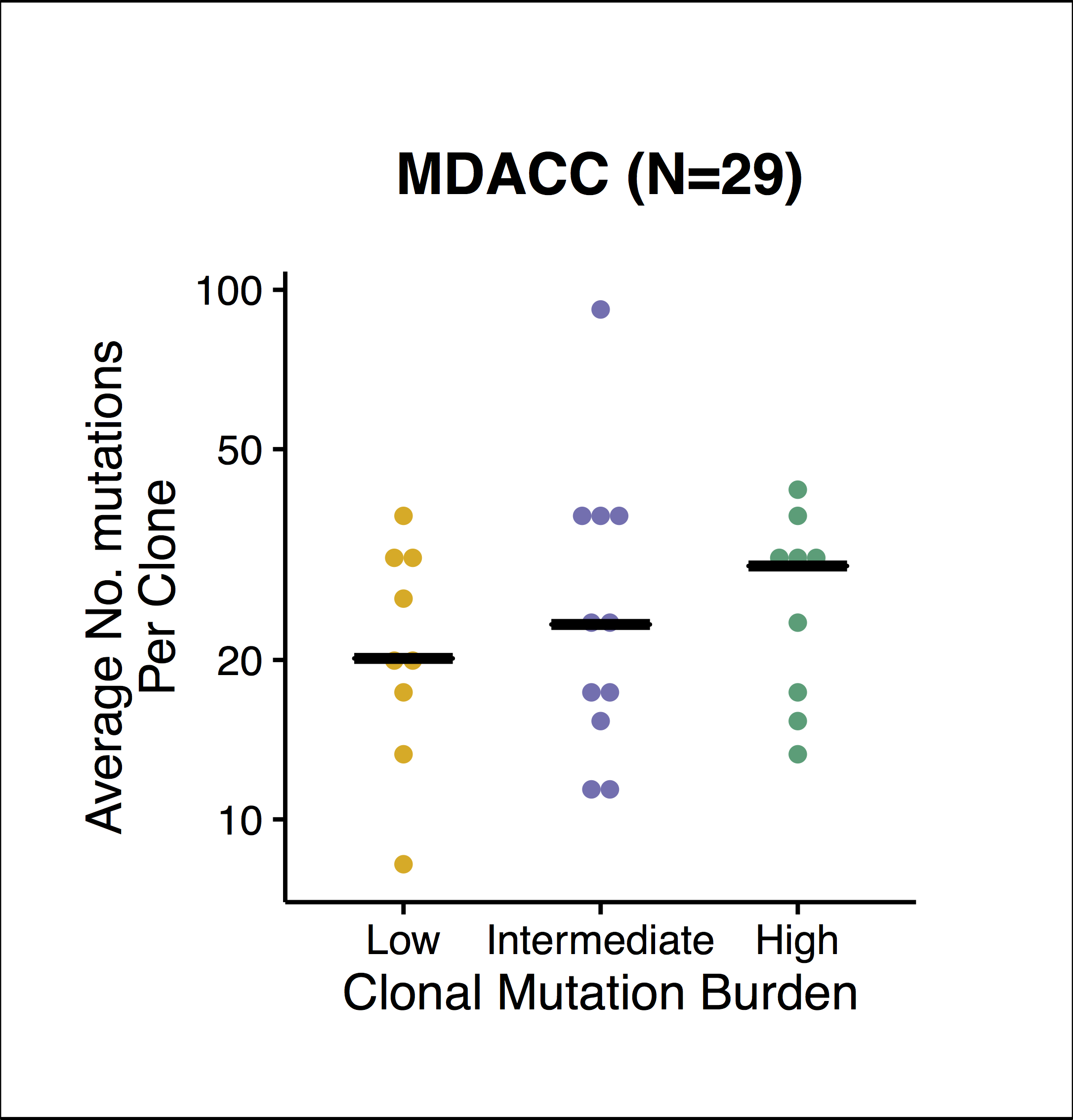

Supplement: S5 Fig — (TIFF) [file pmed.1002193.s006.tiff]

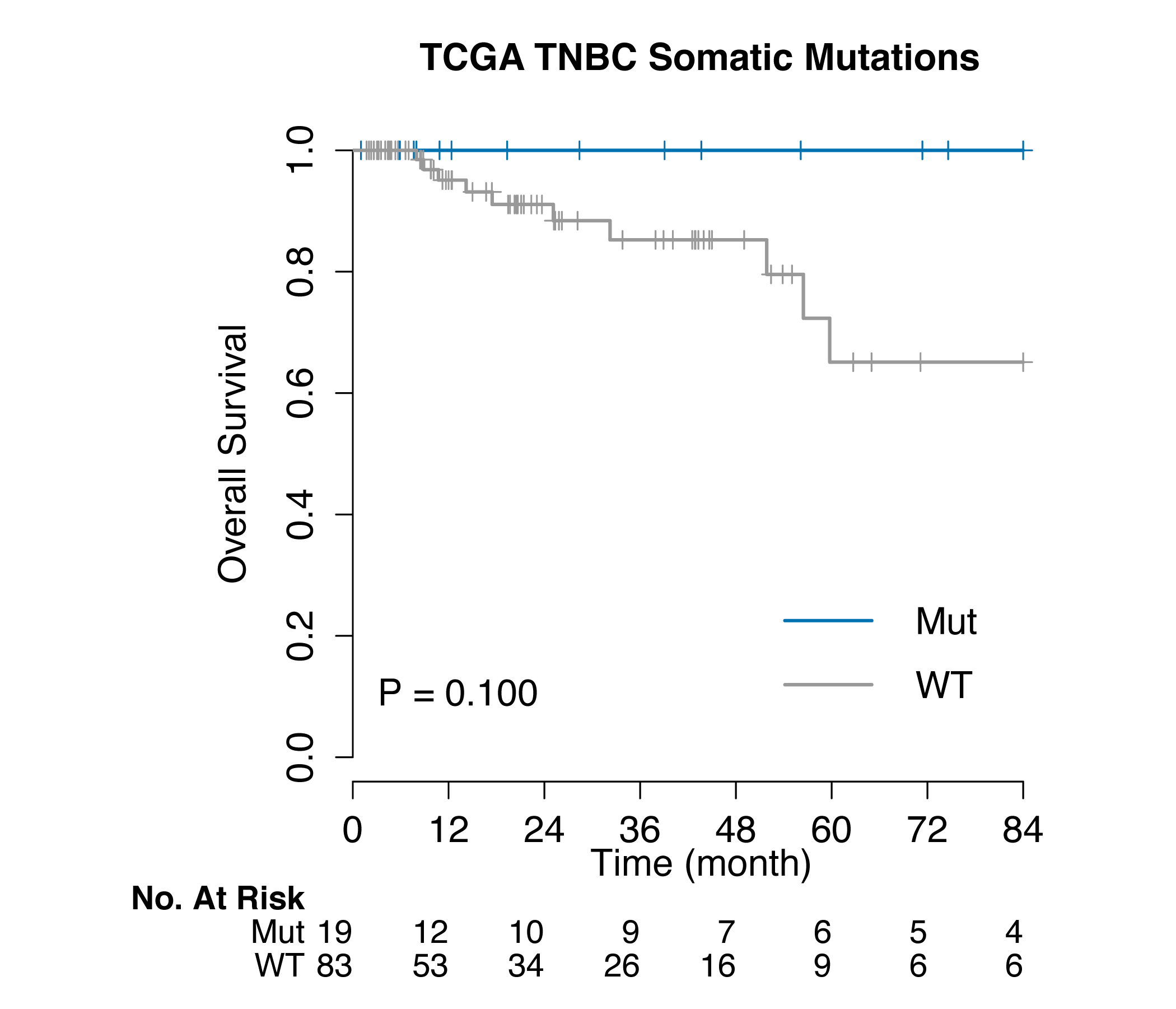

Supplement: S6 Fig — (TIF) [file pmed.1002193.s007.tif]

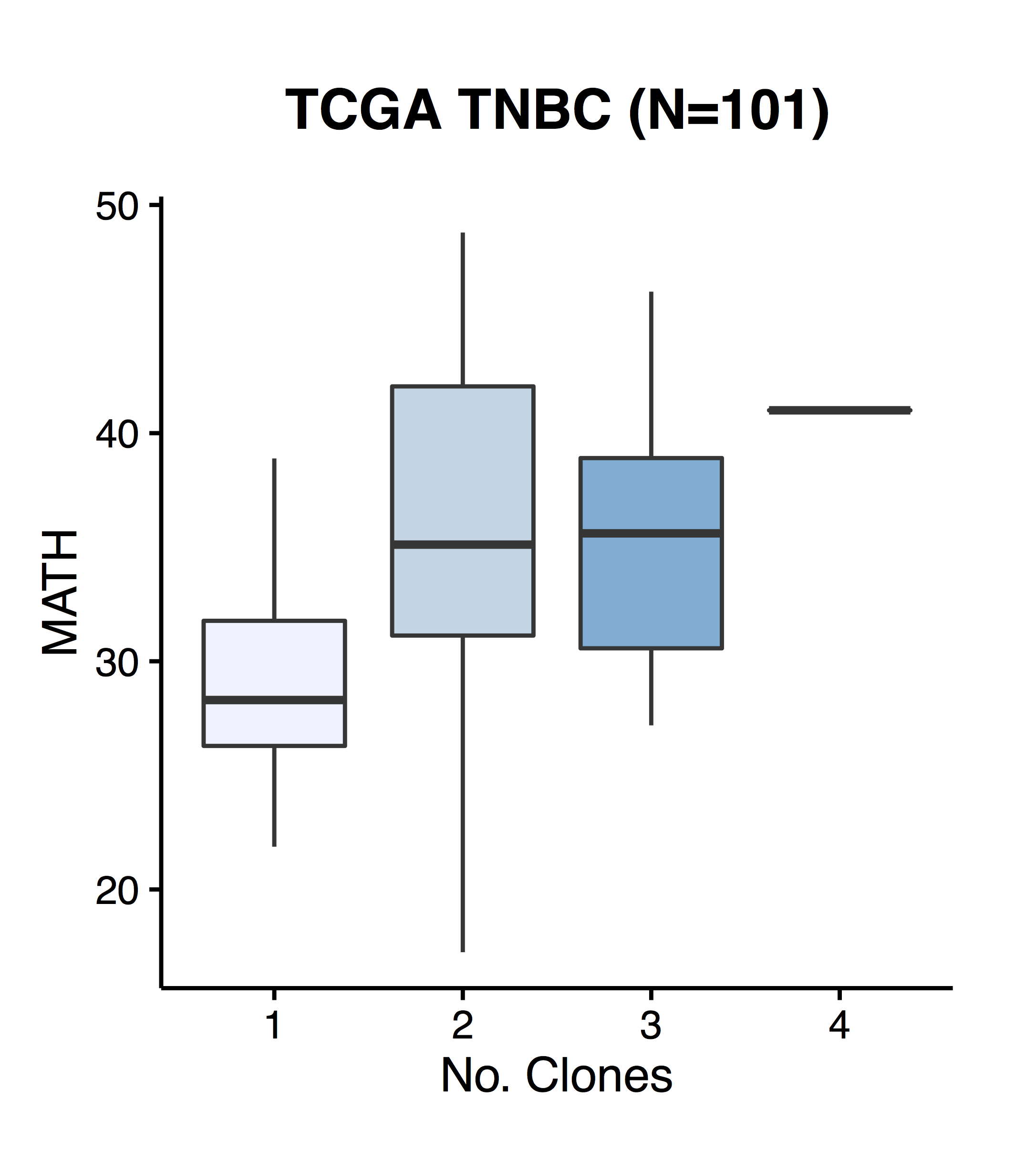

Supplement: S7 Fig — (TIFF) [file pmed.1002193.s008.tiff]

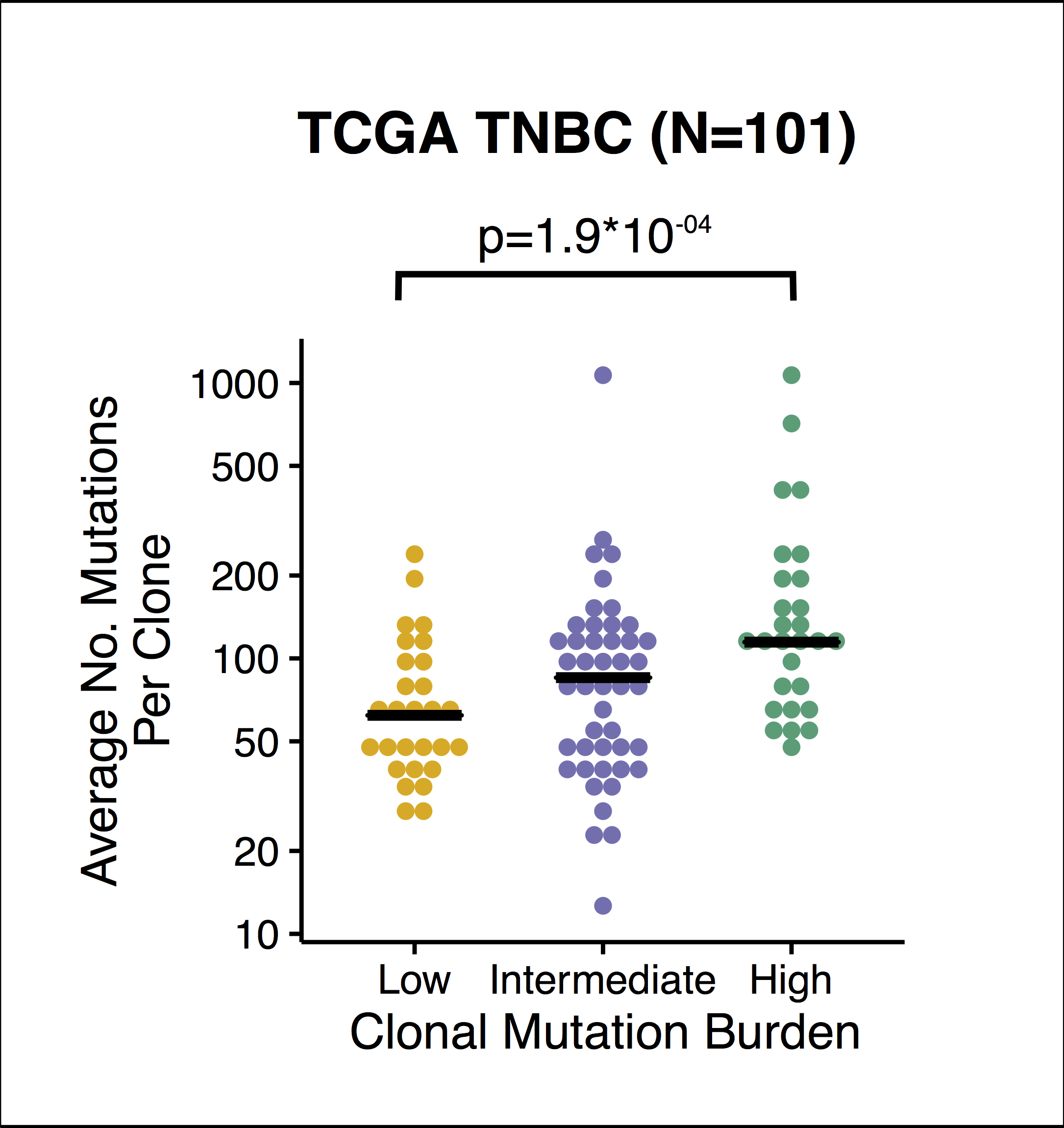

Supplement: S8 Fig — (TIFF) [file pmed.1002193.s009.tiff]

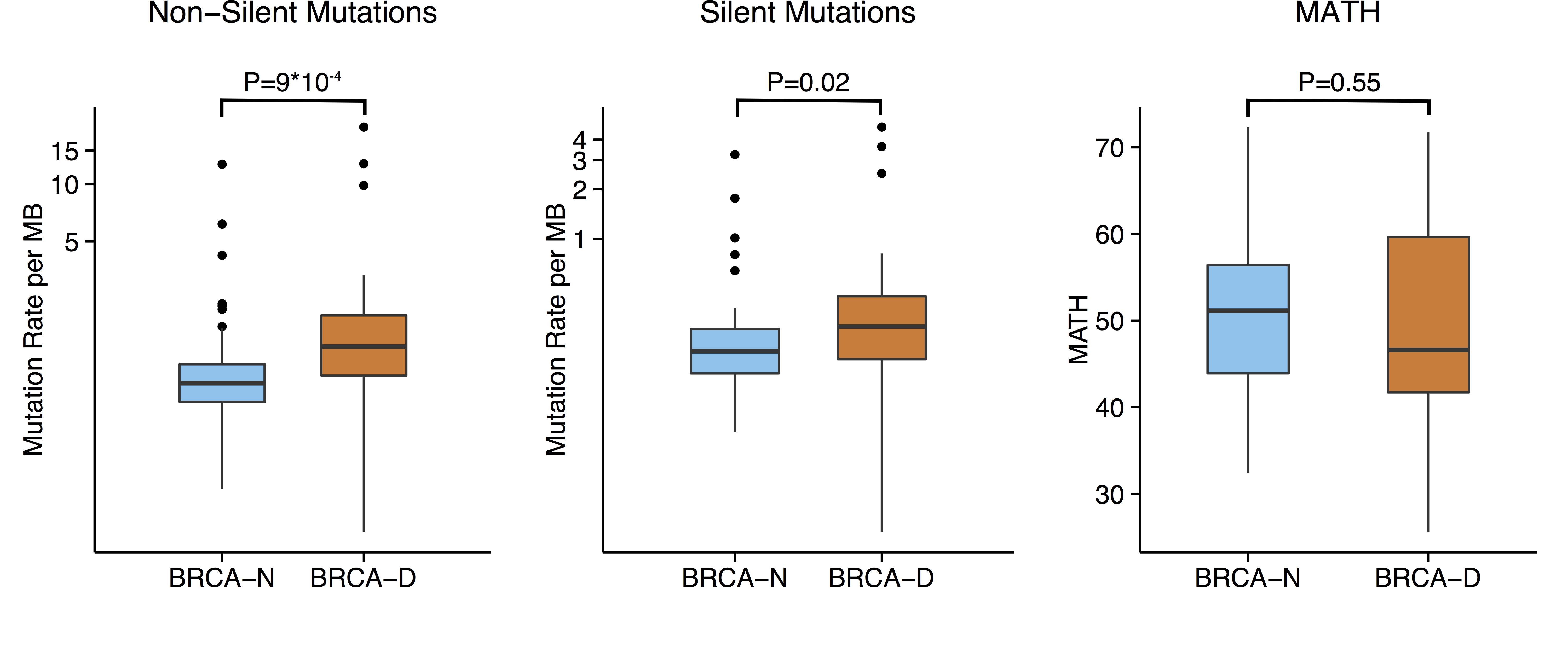

Supplement: S9 Fig — (TIFF) [file pmed.1002193.s010.tiff]

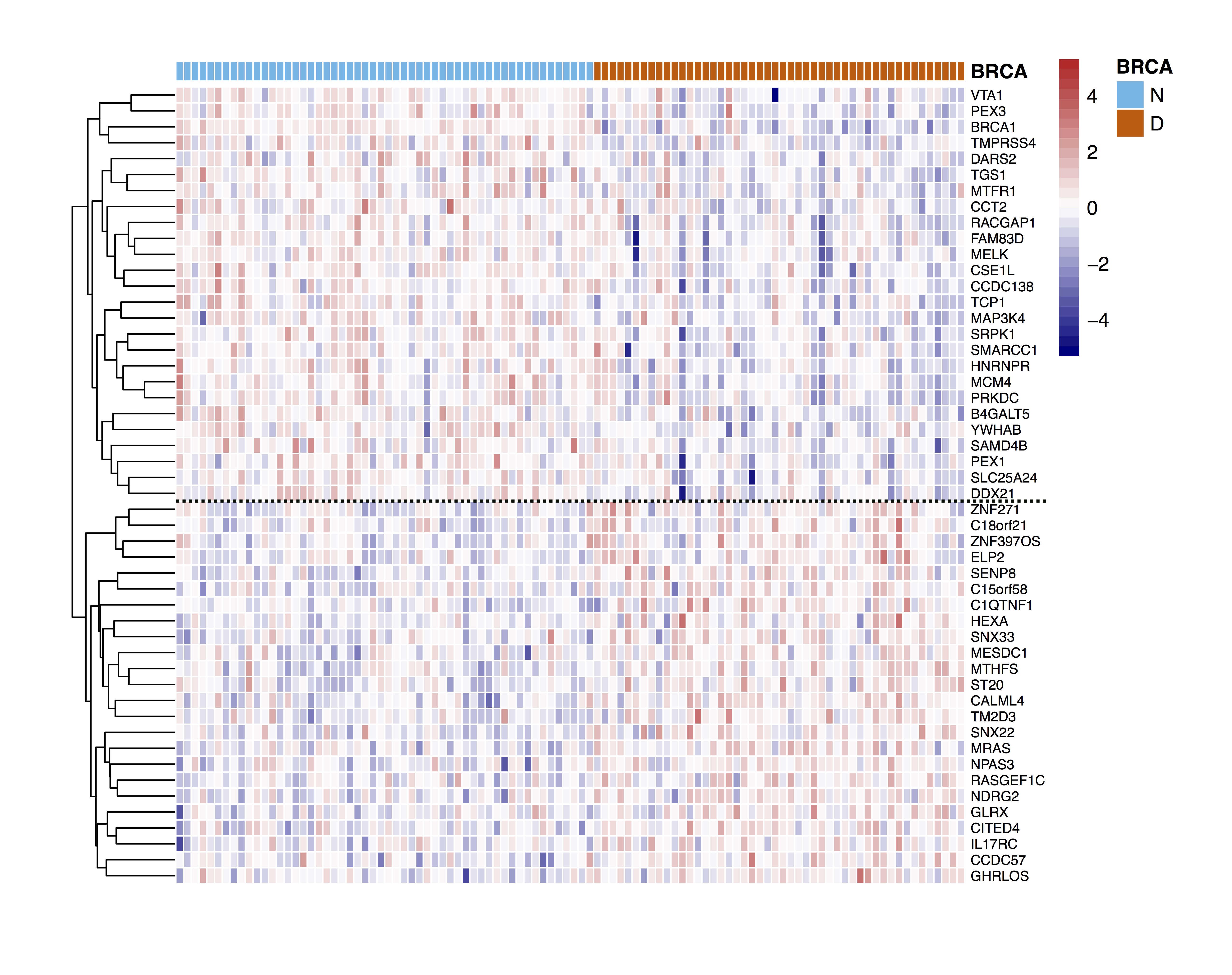

Supplement: S10 Fig — (TIFF) [file pmed.1002193.s011.tiff]

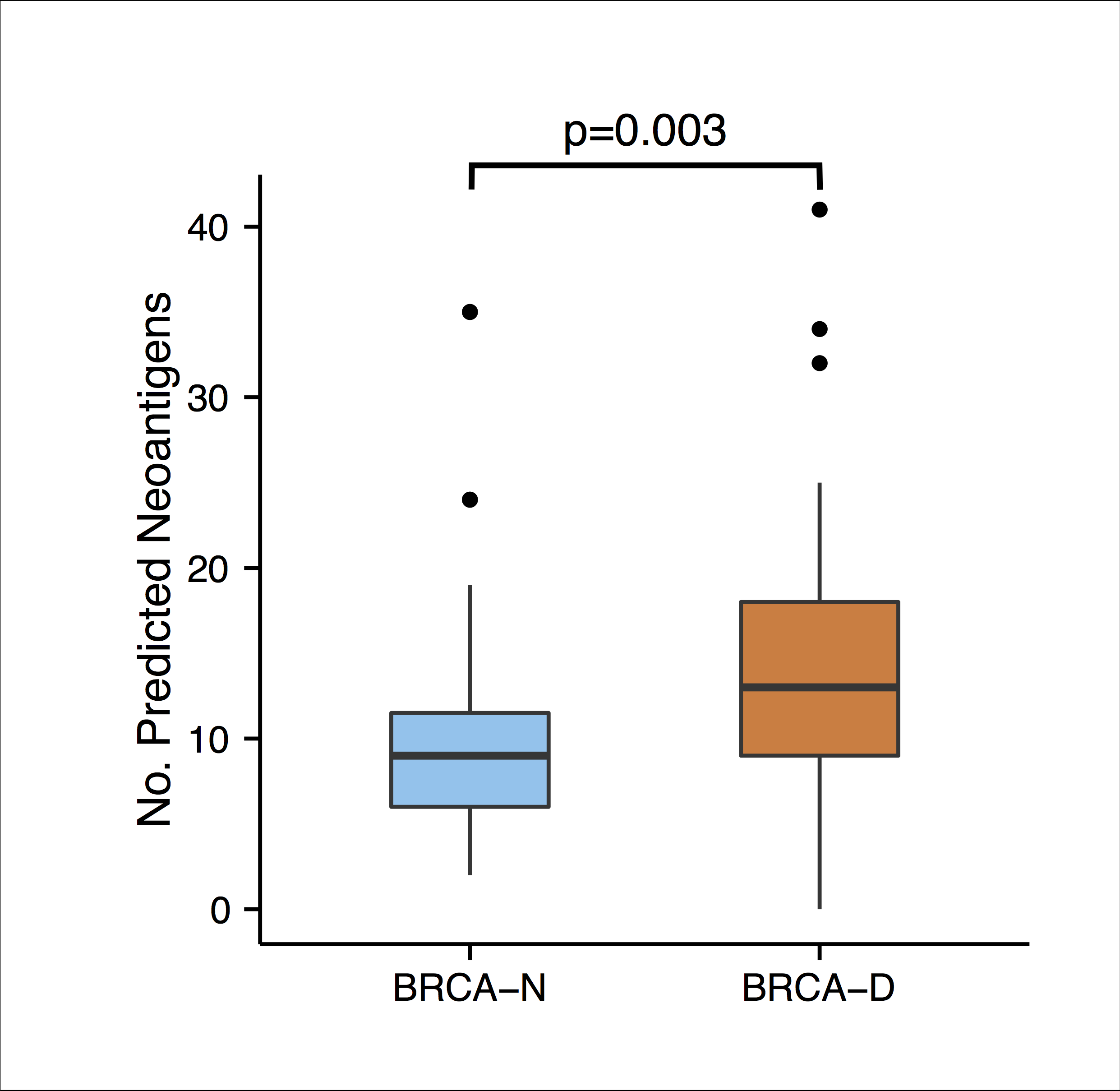

Supplement: S11 Fig — (TIFF) [file pmed.1002193.s012.tiff]

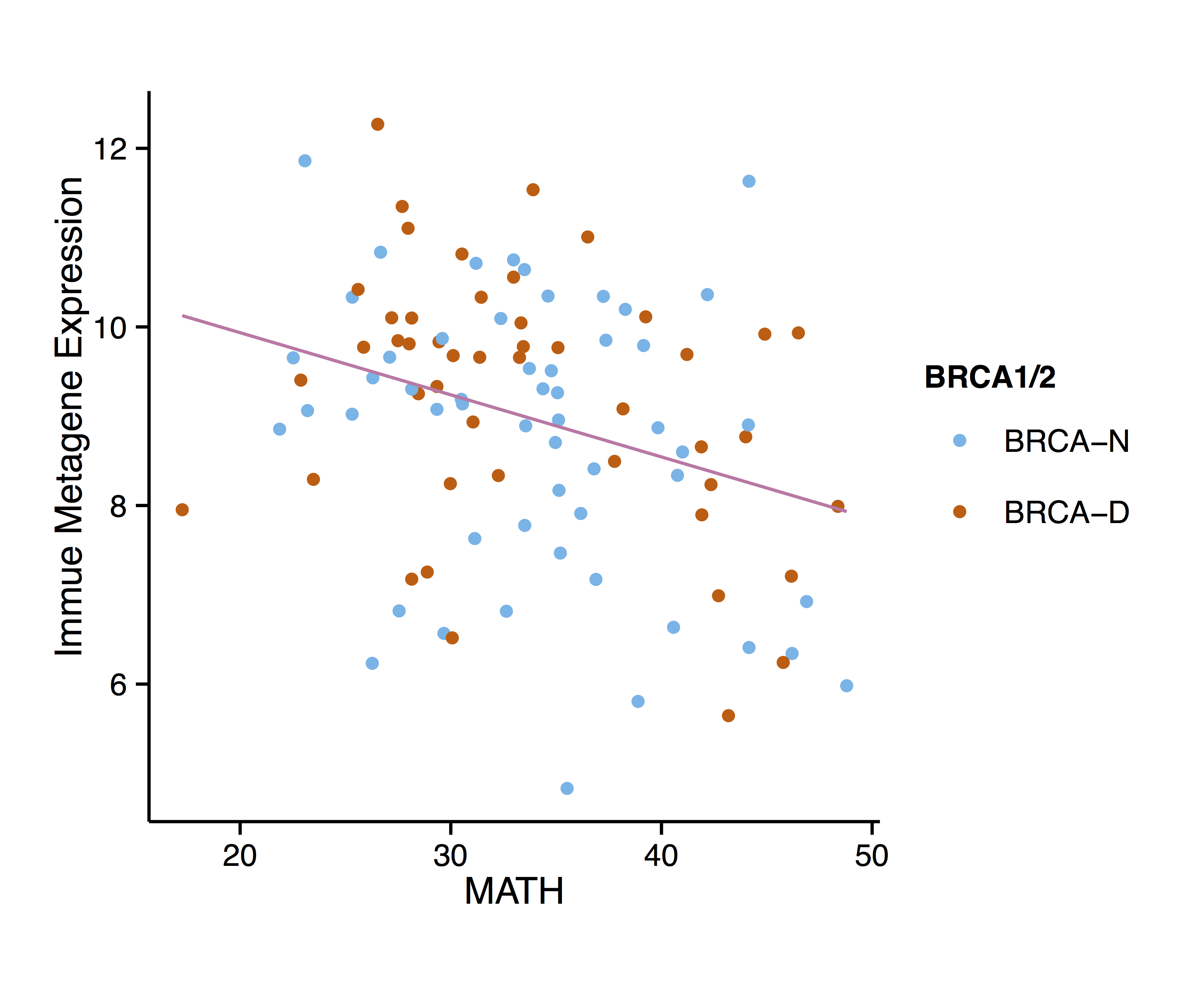

Supplement: S12 Fig — (TIFF) [file pmed.1002193.s013.tiff]
